# Atomically dispersed Lewis acid sites boost 2-electron oxygen reduction activity of carbon-based catalysts

**DOI:** 10.1038/s41467-020-19309-4

**Published:** 2020-10-30

**Authors:** Qihao Yang, Wenwen Xu, Shun Gong, Guokui Zheng, Ziqi Tian, Yujie Wen, Luming Peng, Linjuan Zhang, Zhiyi Lu, Liang Chen

**Affiliations:** 1 Ningbo Institute of Materials Technology and Engineering, Chinese Academy of Sciences, 315201 Ningbo, Zhejiang People’s Republic of China; 2 University of Chinese Academy of Sciences, 100049 Beijing, People’s Republic of China; 3 University of Science and Technology of China, 230026 Hefei, Anhui People’s Republic of China; 4 Key Laboratory of Biomass Chemical Engineering of Ministry of Education, College of Chemical and Biological Engineering, Zhejiang University, 310027 Hangzhou, Zhejiang People’s Republic of China; 5 Fujian Institute of Innovation, Chinese Academy of Sciences, 350002 Fuzhou, Fujian People’s Republic of China; 6 Key Laboratory of Mesoscopic Chemistry of Ministry of Education, School of Chemistry and Chemical Engineering, Nanjing University, 210023 Nanjing, Jiangsu People’s Republic of China; 7 Key Laboratory of Interfacial Physics and Technology, Shanghai Institute of Applied Physics, Chinese Academy of Sciences, 201800 Shanghai, People’s Republic of China

**Keywords:** Catalytic mechanisms, Energy, Density functional theory, Electrocatalysis

## Abstract

Elucidating the structure-property relationship is crucial for the design of advanced electrocatalysts towards the production of hydrogen peroxide (H_2_O_2_). In this work, we theoretically and experimentally discovered that atomically dispersed Lewis acid sites (octahedral M–O species, M = aluminum (Al), gallium (Ga)) regulate the electronic structure of adjacent carbon catalyst sites. Density functional theory calculation predicts that the octahedral M–O with strong Lewis acidity regulates the electronic distribution of the adjacent carbon site and thus optimizes the adsorption and desorption strength of reaction intermediate (*OOH). Experimentally, the optimal catalyst (oxygen-rich carbon with atomically dispersed Al, denoted as O-C(Al)) with the strongest Lewis acidity exhibited excellent onset potential (0.822 and 0.526 V versus reversible hydrogen electrode at 0.1 mA cm^−2^ H_2_O_2_ current in alkaline and neutral media, respectively) and high H_2_O_2_ selectivity over a wide voltage range. This study provides a highly efficient and low-cost electrocatalyst for electrochemical H_2_O_2_ production.

## Abstract (web summary)

H_2_O_2_ production via oxygen reduction offers a renewable approach to obtain an often-used oxidant. Here, authors show the incorporation of Lewis acid sites into carbon-based materials to improve H_2_O_2_ electrosynthesis.

## Introduction

Hydrogen peroxide (H_2_O_2_) is a ubiquitous and environmental friendly oxidant that has seen extensive use for over a century, particularly in applications for bacteria/virus elimination, waste water treatment, paper and pulp bleaching, chemical oxidation, etc^1–3^. Due to the growing population and directly related industrial needs, it is estimated that the global H_2_O_2_ market demand will reach ~6 million tons by 2024^4^. Traditionally, the industrial production of H_2_O_2_ is through the energy-demanding and waste-intensive anthraquinone cycling process. This process requires large-scale production equipment, and further transportation requires handling of large amounts of unstable and hazardous solutions^5^. Thus, the usage of an electrochemical oxygen reduction reaction (ORR) through a 2-electron pathway to produce H_2_O_2_ is a highly desirable method that can be safe, on-site, portable, and green^6–21^. However, this method critically requires the development and screening of low-cost and high-performance electrocatalysts.

Because H_2_O_2_ is the desired final product, the 2-electron ORR catalyst requires that the partial O_2_ reduction preserves the O–O bond and possesses enhanced desorption of the *OOH intermediate at the catalyst surface, unlike the 4-electron ORR process^22^. To meet these key features, the composition and electronic structure of the catalysts are critical and need to be optimized. While it has been shown that the electronic structures of Pt in a PtHg_4_ alloy and Pd in a Pd–Sn alloy are appropriate for 2-electron ORR process^23,24^, the scarcity and high-cost of the metals severely limit their scalability. Carbon-based materials doped with heteroatoms (e.g., nitrogen, oxygen, sulfur), have exhibited promising catalytic activities^25–28^, which can be further improved with transition metal doping to form O–C(M), N–C(M), and S–C(M) structures^29–32^. However, the structure–property relationship at the atomic level remains elusive, and there carry a large risk and uncertainty for these advanced catalyst designs.

In this work, we discover that the 2-electron ORR performance of carbon-based catalysts positively correlates with the Lewis acidity of the dopant. Taking the metal elements in group IIIA (Al and Ga) as examples, our density functional theory (DFT) results indicate that strong Lewis acid sites (octahedral M–O motifs) can enable the activation of neighboring carbon atoms, where the formation and desorption of the key reaction intermediate (*OOH) are facilitated, and excellent catalytic activity for electrochemical H_2_O_2_ production is predicted. To validate the DFT calculations, we rationally fabricate O–C(Al) and O–C(Ga) samples with enriched atomically dispersed Lewis acids via pyrolysis of isostructural channel-type metal-organic frameworks (MOFs, MIL-53)^33–35^. We find that the O–C(Al) with the stronger Lewis acidity exhibits superior activity (onset potential of 0.822 V vs. RHE) and selectivity (>95%) for 2-electron ORR. The proposed correlation is further confirmed by using Cr-doped oxidized carbon materials (O–C(Cr), with moderate Lewis acidity), which expectedly exhibits moderate 2-electron ORR performance. Using electrochemically generated H_2_O_2_ from an O–C(Al) catalyst, we demonstrate an application of bleaching. This work represents a clear demonstration on establishing the relationship between the Lewis acidity with the electrocatalytic ORR activity in carbon-based catalysts.

## Results

### Theoretical calculations

**Fig. 1:**
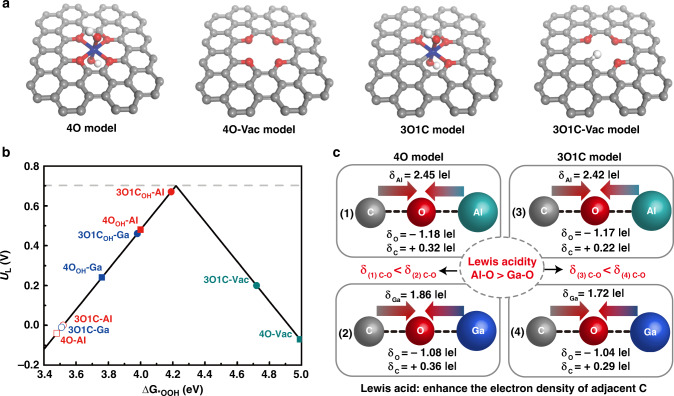
The atomically dispersed Lewis acid in an oxygen-rich carbon catalyst enhanced the catalytic performance of the 2-electron ORR reaction. **a** Four DFT calculation models with potential active sites for electrochemical production of H_2_O_2_. Blue, red, white, and gray balls denote metal, O, H, and C atoms, respectively. **b** The simulated activity volcano plot for 2-electron ORR path to H_2_O_2_. The theoretical equilibrium potential is shown as a gray dashed line. **c** Illustration showing the effect of Lewis acidity to the electron distribution of adjacent carbon atom.

As the aluminum compound is a commonly used strong Lewis acid^36^, we chose the metal elements in group IIIA (Al, Ga, and In) as the research targets. It is qualitatively demonstrated that the Lewis acidity increases as the atomic number of elements decrease if the centers of Lewis acids are in the same main group with the same chemical environment. DFT calculations were first performed to screen possible candidates that can achieve a high electrocatalytic performance of the 2-electron ORR^25,30^. We constructed graphene sheet-based models with various coordination environments for simulation, as shown in Fig. 1a and Supplementary Fig. 1. However, due to the weak coordination ability of In, the simulated octahedral structures containing In were found to be thermodynamically unstable, and thus were not investigated in detail. Additional hydroxyl groups were connected to the metal center to construct saturated octahedral or tetrahedral coordination environments. We considered two types of coordination oxygen in the graphene sheet, labeled as terminal oxygen and ether oxygen, respectively. However, the geometry optimizations of key intermediates in the ORR process show that the structures containing terminal oxygen are so active that the neighboring carbon sites bind to the oxygen-containing group intensely, leading to substrate deformation and collapse. This is understandable because these phenol-like structures are quite reactive in organic chemistry. In contrast, the ether group is stable even at high temperature, and thus the α carbon atoms adjacent to the ether oxygen are supposed to be the active sites for H_2_O_2_ generation^25,37^. Accordingly, we mainly focused on two models labeled as 4O and 3O1C. For comparison purposes, the corresponding structures without metal dopants were also simulated (denoted as 4O-Vac and 3O1C-Vac, respectively). It is well accepted that the activity of 2-electron ORR is determined by either the step of O_2_ activation (Eq. (1)) or desorption of the *OOH intermediate (Eq. (2)):

1 $$\ast + {\mathrm{O}}_2 + {\mathrm{H}}_2{\mathrm{O}} + {{e}}^ - \to \ast {\mathrm{OOH}} + {\mathrm{OH}}^ -,$$

2 $$\ast {\mathrm{OOH}} + {{e}}^ - \to {\mathrm{HO}}_2^ - + \ast.$$

Herein, based on the computational hydrogen electrode (CHE) model, the commonly used descriptor, ΔG_*OOH_, was calculated to evaluate the 2-electron ORR activity of each site (Fig. 1b and Supplementary Table 1)^25,30,32^. Firstly, our calculations suggest that the binding strength of O_2_ on α carbon atoms in each Vac model without metal dopants is relatively weak, in agreement with previous studies^25,30^. Correspondingly, the calculated ΔG_*OOH_ resides on the right leg of the volcano plot and is located far away from the summit, indicating that these carbon sites only have moderate activity (Fig. 1b). On the contrary, the carbon atoms coordinated with metal dopants (octahedral Al–O and Ga–O) can significantly activate the hydrogenation of O_2_. For an electrochemical reaction that occurs at the interface, enhancing the interaction of gas to the surface can significantly improve the reaction kinetics. On the other hand, the most favorable adsorption sites of metal-doped systems bind to *OOH so intensely that the H_2_O_2_ formation is hindered (as shown at the lower left of the volcano plot in Fig. 1b). Accordingly, these sites might be deeply oxidized to generate *OH. Herein we further located the corresponding *OH intermediates and calculated free energy change of the *OH desorption step at 0.70 V. The positive desorption energies (Supplementary Table 2) implies that *OH can hardly be desorbed under the experimental conditions. These sites that bind *OOH too strongly would be occupied by *OH during the oxidation process, similar to the high *OH coverage of oxidized metal surface in the ORR process (Supplementary Fig. 2)^38^. Instead, the sites that bind *OOH relatively less strongly can achieve optimal ΔG_*OOH_’s. The Al-doped models with stronger Lewis acidity possess better activities for 2-electron ORR than Ga-doped models. For the 4O and 3O1C models, the overpotentials of Al-doped systems are only 0.03 and 0.22 V, respectively. Besides, we explored the selectivity of the 2-electron process over the 4-electron process. As suggested by Guo et al.^39^, for the electrocatalysts favoring the generation of H_2_O_2_ rather than H_2_O, the potential barrier for H_2_O_2_ desorption should be lower than that for the hydrogenation of OOH* to O*. The barriers are listed in Supplementary Table 3, where one can see that the 2-electron process is thermodynamically favorable or comparable to the 4-electron process. Considering the fact that the 2-electron process is also kinetically preferred, we believe that the 2-electron process should be dominant.

Furthermore, Bader charge analysis was employed to elucidate the effect of the metal centers (Fig. 1c). The well-established theory on Lewis acid catalysis demonstrates that metal atoms can coordinate with an electronegative atom with the lone-pair, resulting in charge transfer and a more electronegative lone-pair donor^40^. In the 4O-Vac model, the ether oxygen and the potentially active carbon possess atomic charges of −0.98 and +0.65|*e*|, respectively, which means that electrons accumulated on the C–O bonds were −0.33|*e*|. In comparison, Lewis acid coordination allows for electron transfer from the metal to the C–O bond, resulting in a more negative charge on the oxygen and a less positive charge on the α carbon. In the 4O model, the charges on oxygen and α carbon are −1.18 and +0.32|*e*| in the Al-containing system, while in a Ga-containing system, they are −1.08 and +0.36|*e*|, respectively. Therefore, more electrons can be accumulated on the C–O bonds (−0.86 and −0.72|*e*| for Al-containing and Ga-containing systems, respectively) once Lewis acid sites are introduced. The higher electron accumulation or density on the C–O bond (i.e., carbon site with a less positive charge) can enhance the interaction between O_2_ molecules and α carbon, and thus facilitate the formation of *OOH intermediates. Similarly, in the 3O1C model, the carbon atom directly binds to the metal center and withdraws electrons, resulting in higher electron density for the carbon atom. Since the Lewis acidity of Al-center is stronger than that of Ga-center, 3O1C–Al yields an even more negative atomic charge [−0.95 and −0.75|*e*| for O–C(Al) and O–C(Ga), respectively] and higher activity for *OOH formation. Moreover, we further depict the deformation charge density to illustrate the charge transfer between the oxygen-doped carbon layer and metal motif (Supplementary Fig. 3). It is clearly seen that the electrons tend to accumulate on the α carbon after coordinating to Al or Ga, also consistent with the atomic charge analysis. From the perspective of electronic structure, the effect of functional motif has already been demonstrated both experimentally and theoretically. It was reported that the nitrogen dopant in carbon materials withdraws electron from the adjacent C atom, leading to a reduced work function, increased density of π states close to Fermi level^41^. As a result, the 4-electron ORR process is promoted. In contrast, the high work function of oxygen-doped porous carbon is related to the dominant 2-electron process^12^. Introducing Lewis acid that coordinates to oxygen may further result in electron accumulation on active site and consequently promote the 2-electron process. These properties, including acidity/basicity, work function, and density of states near the Fermi level, are all correlated with charge density. Moreover, due to electrostatic interaction, the binding strength between negatively or less positively charged carbon and generated electronegative *OOH can be weakened, thereby enhancing the desorption of *OOH. As a result, the carbon atom binding to Al may exhibit the highest performance of the 2-electron ORR reaction. Indeed, as shown in the activity volcano plot (Fig. 1b), the potential active sites in the Al-doped system are located closer to the volcano summit than those in the Ga-doped system.

### Synthesis and characterizations of O–C(M) catalysts

**Fig. 2:**
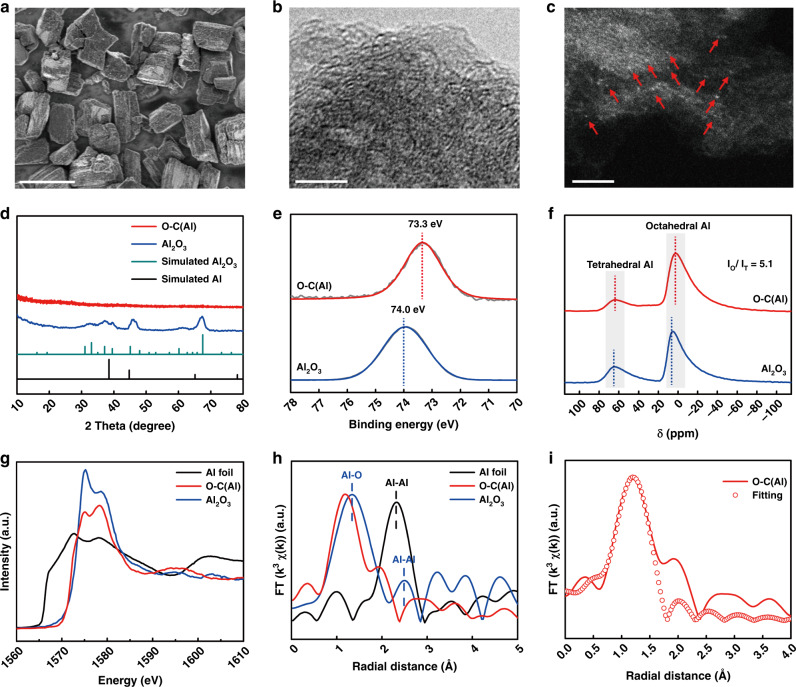
Synthesis and structural characterization of O–C(M). **a** SEM image of O–C(Al), scale bar is 5 μm. High-resolution TEM (**b**) and aberration-corrected HAADF-STEM (**c**) images of O–C(Al), scale bars are 5 and 2 nm, respectively. **d** Powder XRD patterns for simulated Al and Al_2_O_3_, as-synthesized O–C(Al) and commercial Al_2_O_3_. **e** High-resolution XPS spectra of Al 2p in O–C(Al) and Al_2_O_3_. **f** Al solid-state NMR patterns of O–C(Al) and Al_2_O_3_. **g** Al K-edge XANES and **h** k^3^-weighted Fourier transform of EXAFS spectra. **i** The EXAFS R-space fitting curve of O–C(Al).

Two isomorphic MOFs, MIL-53(Al, Ga), with enriched octahedral M–O clusters were chosen as the representative precursors. Powder X-ray diffraction (XRD) patterns and scanning electron microscopy (SEM) images show the successful synthesis of isomorphic MIL-53(Al, Ga) with highly crystalline and regular morphology (Supplementary Figs. 4 and 5). Upon a pyrolysis (800 °C for 2 h in N_2_ atmosphere) and room temperature chemical etching process (immersed in 0.1 M NaOH for 12 h), the O–C(M) catalysts with atomically dispersed Lewis acids sites (Al–O and Ga–O species) were prepared with highly maintained original morphologies (Fig. 2a and Supplementary Fig. 6).

Further characterization was necessary to elucidate how the structure of the catalysts impacts or determines the catalytic performance. From the DFT studies, we first studied O–C(Al), as it was predicted to be the best catalyst for 2-electron ORR. Elemental mapping at the microstructural level via SEM indicated that Al and O were uniformly distributed in the carbon matrix (Supplementary Fig. 7) for O–C(Al), indicating that the MOF precursor has an equidistribution effect. High-resolution transmission electron microscopy (TEM) images (Fig. 2b) also proved that there were no identifiable Al or Al_2_O_3_ nanoparticles in O–C(Al) sample, while aberration-corrected high angle annular dark-field scanning TEM (HAADF-STEM) observations demonstrated that the Al species were atomically dispersed within the carbon matrix (Fig. 2c), consistent with XRD analysis (Fig. 2d). Inductively coupled plasma (ICP) results also showed that the content of the Al species was as high as 5.85 wt% in O–C(Al). Similarly, the characterizations showed that there were many atomically dispersed Ga species (Supplementary Fig. 8) in O–C(Ga). However, there is a small amount of Ga_2_O_3_ that remains even after chemical etching (Supplementary Fig. 9), which may arise from the protection of carbon layers on the surface of Ga_2_O_3_.

More critically, we used X-ray photoelectron spectroscopy (XPS) measurements to determine the binding energy and chemical signatures of the Al species in the O–C(Al) sample. XPS revealed the presence of Al, O, and C (Supplementary Fig. 10) expectedly, with a high-resolution single-peaked Al 2*p* spectrum at a binding energy of 73.3 eV (Fig. 2e). It should be noted that this peak position was located between that of Al_2_O_3_ (74.0 eV) and Al (72.7 eV)^42,43^, illustrating that the valence state of the Al species in O–C(Al) was between Al^0^ and Al^3+^. Compared with O–C sample, the high-resolution XPS spectra of C 1*s* and O 1*s* in the O–C(Al) catalyst demonstrated that the binding energy of O and C atoms adjacent to Al atom were both shifted to lower binding energy (Supplementary Fig. 11), which clearly indicated that more electrons were accumulated on the O and C atoms adjacent to Al atom, consistent with the computational analysis of deformation charge density (Supplementary Fig. 3) and Bader charge (Fig. 1c). Nuclear magnetic resonance (NMR) was used as an additional method to determine the coordination of the Al species, as it is sensitive to the coordination environment. The solid-state NMR of Al yielded two peaks centered at 3 and 63 ppm, which can be assigned to octahedral and tetrahedral Al–O species, respectively (Fig. 2f)^44^. The high-intensity ratio (*I*_O_/*I*_T_ = 5.1) of the octahedral band and tetrahedral band also indicated that the octahedral Al–O species was predominant. In order to confirm the structure of O–C(Al) at the atomistic level, X-ray absorption spectroscopy (XAS), a powerful technique to determine the coordination environment and valence state of the target atoms was employed. The X-ray absorption near-edge structure (XANES) spectra of O–C(Al) in Fig. 2g illustrated that the valence state of Al species in O–C(Al) was situated between that of Al^0^ and Al^3+^, consistent with the afore-mentioned XPS result. Moreover, the result can be further verified by the differential curves obtained from the XANES data (Supplementary Fig. 12). The extended X-ray absorption fine structure (EXAFS) spectra for O–C(Al) exhibited the main peak at ~1.2 Å, which could be attributed to Al–O(C) scattering paths. Importantly, the fingerprinting signal peaks of Al–Al interactions in Al foil (~2.3 Å) and Al_2_O_3_ (~2.5 Å) cannot be observed in the curve of O–C(Al)^45^. Therefore, it can be concluded that Al atoms were atomically dispersed in O–C(Al). Furthermore, the best fitting result of the obtained EXAFS data reveals that the coordination number of Al was about 6 (Fig. 2i and Supplementary Table 4), which is in agreement with the result of solid-state NMR of O–C(Al) and the constructed model structure in DFT calculations. Unfortunately, the XAS data cannot directly prove the existence of Al–C as the bond length of Al–C and Al–O are very close. Thus, all characterizations suggest that the pyrolysis of the MOF, MIL-53(Al), were successful in the fabrication of carbon-based catalyst featuring atomically dispersing the metal species in an octahedral environment with valences that are predicted to regulate the electron distribution of α carbon atom and in turn positively affect the 2-electron ORR.

### Electrocatalytic 2-electron ORR characterizations

**Fig. 3:**
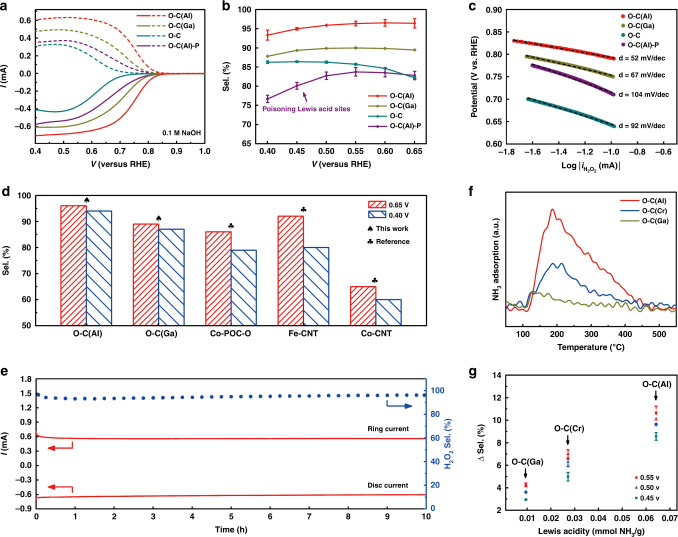
Electrocatalytic performance of O–C(M) and O–C towards 2-electron ORR in 0.1 M NaOH. **a** Illustration showing the process of O_2_ reduction to H_2_O_2_ over O–C(M) catalysts. Linear sweep voltammetry (LSV) curves of O–C(Al), O–C(Ga), O–C, and O–C(Al)–P (solid lines) together with the corresponding H_2_O_2_ currents on the ring electrode (dashed lines) recorded at 1600 rpm. **b** Calculated H_2_O_2_ selectivity during the potential sweep. **c** Tafel slopes of O–C(Al), O–C(Ga), O–C, and O–C(Al)–P in 0.1 M NaOH solution. **d** The H_2_O_2_ selectivity of O–C(M) and reference catalysts at low overpotential (0.65 V) and high overpotential (0.4 V). **e** Stability measurement of O–C(Al) at a fixed disk potential of ~0.47 V. **f** NH_3_-TPD profiles of O–C(Al), O–C(Cr), and O–C(Ga). **g** The correlation between the increased selectivity (compared with O–C catalyst) and Lewis acidity of O–C(M) catalysts at various potentials.

To determine the performance of the O–C(M) catalysts, the ORR activity and selectivity of all electrocatalysts were evaluated on a rotating ring-disk electrode with a collection efficiency of 0.35 (calibrated by the redox reaction of [Fe(CN_6_)]^4−^/[Fe(CN_6_)]^3−^, Supplementary Fig. 13) in 0.1 M NaOH (Fig. 3a, b). The disk electrode and Pt ring electrode were responsible for the O_2_ reduction current (solid line) and H_2_O_2_ oxidation current (dashed line), respectively. The ORR can undergo either a 2-electron or 4-electron pathway, while the former pathway is preferred for all O–C(M) catalysts. An ultrahigh onset potential (0.822 V at 0.1 mA cm^−2^ H_2_O_2_ current) and selectivity (>95%) within the potential range of 0.45–0.65 V vs. RHE were observed for the O–C(Al) catalyst, as shown in Fig. 3a, b. It should be noted that this onset potential is superior to those of so-far reported H_2_O_2_ catalysts (Supplementary Table 5). Moreover, the Tafel Slope of O–C(Al) sample (~52 mV dec^−1^) is lower than other O–C(M) samples, indicating its faster reaction kinetics. We have also tuned the pyrolysis temperature (700–900 °C) to synthesize O–C(Al) catalysts and found that temperature did not affect the high catalytic performance of the 2-electron ORR using the O–C(Al) catalyst, demonstrating its robustness and reproducibility (Supplementary Figs. 14 and 15). Compared with the O–C(Al) catalyst, the O–C(Ga) catalyst exhibited slightly decreased onset potential, selectivity, and Tafel slope (Fig. 3a–c). The proposed correlation not only is applicable for main group metals but also can be further extended to transition metals. An O–C(Cr) catalyst (detailed characterizations can be found in Supplementary Fig. 16) with moderate Lewis acidity possessed moderate ORR performance (Supplementary Fig. 17), demonstrating the effectiveness of constructing atomically dispersed M–O Lewis acid sites on electrochemical oxygen reduction to H_2_O_2_ production. While transition metal-doped carbon materials have already been identified as highly active catalysts for 2-electron ORR, the previously reported transition metal sites (e.g., Co, Fe) in the catalysts may serve as the active sites for 4-electron ORR^46,47^ and the high selectivity cannot be maintained under high overpotentials (Fig. 3d and Supplementary Table 6)^30,48^. On the contrary, main group metals, such as Al and Ga in this work, are thought to be catalytically inactive due to the tight binding of the metal center with the hydroxyl group intermediate that leads to the suppression of the intermediate product desorption. The oxygen-rich carbon catalyst (preparation in methods) without M–O clusters exhibited poor catalytic performance (onset potential of 0.698 V and selectivity of ~85%), unequivocally indicating the significant role of Lewis acid sites in enhancing the reduction of O_2_ to H_2_O_2_. More importantly, the activity and selectivity can be well maintained during long-term testing (~10 h), indicating the excellent recyclability and the stability of O–C(Al) (Fig. 3e).

As M_2_O_3_ (M = Cr, Ga) and atomically dispersed M–O sites coexisted in the O–C(M) catalysts, we measured the ORR performance of pure M_2_O_3_ to exclude their possible contribution. The selectivity of Cr_2_O_3_ and Ga_2_O_3_ was found to be only ~58% and ~61% (Supplementary Fig. 18), respectively, revealing their inert nature and negligible contribution for the 2-electron ORR. This is further proof that the high ORR activity and selectivity of O–C(Cr) and O–C(Ga) catalysts can be attributed to the atomically dispersed Cr/Ga species rather than the M_2_O_3_ nanoparticles covered in the carbon matrix.

Encouraged by the improved activity of O–C(M), group IIIA metal elements (Al and Ga) doping has been attempted to enhance the performance of other carbon-based catalysts, such as nitrogen-rich and sulfur-rich carbon (denoted as N–C(M) and S–C(M), respectively). As expected, in both N–C(M) and S–C(M) system, the H_2_O_2_ selectivity of M-doped (M = Al, Ga) carbon was higher than that of no metal-doped carbon, and Al-doped catalyst with stronger Lewis acidity exhibited superior H_2_O_2_ selectivity (Supplementary Figs. 19 and 20), which was in good agreement with the results of DFT calculations (Supplementary Fig. 21 and Supplementary Table 7). All results unambiguously demonstrate that group IIIA metal (Al and Ga) doping is an effective and universal strategy to improve the 2-electron ORR performance of carbon-based catalysts and the H_2_O_2_ selectivity of catalysts is positively correlated with the Lewis acidity of the dopant.

Another method we used to prove that the Lewis acidity is crucial in the 2-electron ORR activity was through pyridine poisoning. As pyridine’s lone-pair has the ability to strongly coordinate with unoccupied orbitals of transition metals, it can fully poison the catalytic activity of the Lewis acid sites^49^. Once the atomically dispersed Lewis acid sites (octahedral Al–O) in O–C(Al) were effectively poisoned by pyridine (denoted as O–C(Al)–P), the catalytic H_2_O_2_ selectivity was sharply reduced to ~80% (Fig. 3c), close to that of the Vac, O–C sample. The Lewis acidity of all electrocatalysts was measured via the temperature-programmed desorption of ammonia (NH_3_-TPD), which is reliant on the binding strength between the adsorbed NH_3_ and the adsorption sites (Fig. 3f)^50–52^. Generally, the adsorption site with stronger acidity presents a stronger binding strength with NH_3_ and requires higher temperatures for NH_3_ desorption. The results demonstrated that O–C(Al) exhibited the strongest Lewis acidity (i.e., Al in O–C(Al) in a state of less electrons) while O–C(Ga) exhibited the weakest Lewis acidity (Supplementary Fig. 22). More valence electrons (outermost electrons) of aluminum in O–C(Al) were delocalized to the surrounding oxygen-rich carbon (O–C) substrate, which is consistent with the Bader charges analysis (Fig. 1c). The selectivity of reducing O_2_ to H_2_O_2_ over O–C(M) is closely related to its acidity, where Fig. 3g demonstrates a nearly linear correlation between the increased selectivity (Δ sel., compared with O–C catalyst) and Lewis acidity, which is in good agreement with the conclusions from DFT calculations. Compared with the O–C system, integration of the Lewis acid sites (M–O) into the oxygen-rich carbon to form O–C(M) can effectively improve the formation of the key intermediate (*OOH), which is critical for the 2-electron ORR reaction (Fig. 1b). However, the excessively strong adsorption strength will affect the desorption of *OOH and decrease the selectivity of the reaction. The O–C(M) with the stronger Lewis acidity means that more valence electrons from the metal centers are donated to the neighboring carbon atoms, increasing the electron density of the carbon atoms (Fig. 1c). The rich-electron structure is conducive to partially reduce the over-binding strength to make the ΔG_*OOH_ closer to the theoretical equilibrium potential. As a result, the catalytic performance of the catalyst towards 2-electron ORR reaction is gradually improved along with the increasing of the Lewis acidity of the catalyst and is arranged in the followed order of O–C(Ga) < O–C(Cr) < O–C(Al).

**Fig. 4:**
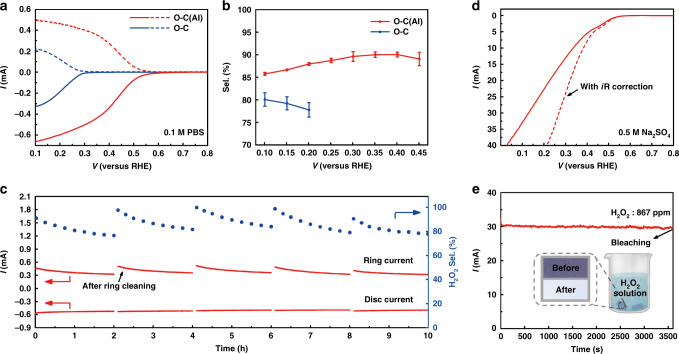
ORR performance of O–C(Al) and O–C in neutral neutral electrolyte. **a** LSV curves of O–C(Al) and O–C (solid lines) together with the corresponding H_2_O_2_ currents on the ring electrode (dashed lines) recorded at 1600 rpm in 0.1 M PBS. **b** Calculated H_2_O_2_ selectivity at different potentials. **c** Stability measurement of O–C(Al) at a fixed disk potential of ~0.2 V. The ring electrode was refreshed by rapid scan at negative potentials every two hours. **d** LSV of O–C(Al) catalyst in 0.5 M Na_2_SO_4_ via a H-cell electrolyzer. **e** Bulk electrolysis at a constant current density of 30 mA cm^−2^ in 0.5 M Na_2_SO_4_. Insert: the bleaching demonstration of blue litmus paper with the generated H_2_O_2_ (~867 ppm).

This enhancement of catalytic performance over oxygen-rich carbon with atomically dispersed Lewis acid sites was also observed in a neutral electrolyte (0.1 M phosphate-buffered saline, PBS, pH ~7). Compared with O–C (selectivity: ~75%; onset potential: 0.266 V vs. RHE at 0.1 mA cm^−2^ H_2_O_2_ current), H_2_O_2_ generation was significantly improved with the O–C(Al) catalyst, among which the onset potential shifted in a positive direction by ~260 mV (0.526 V vs. RHE) and the selectivity increased to ~90% (Fig. 4a, b). The stability of the O–C(Al) was also examined under long-term testing (~10 h), in which the remarkable changes in activity and selectivity cannot be measured (Fig. 4c). The gradual decay of the ring current in PBS was caused by the anion poisoning rather than the degradation of current, which can be restored after cleaning.

In addition, practical electrolysis was demonstrated in an H-cell, where the O–C(Al) catalyst was casted onto a carbon paper with the loading amount of 1 mg cm^−2^. The result shows that the current density of the catalyst is significantly improved compared with that in the rotating ring-disk electrode and the selectivity above 90% (determined by a colorimetric quantification method) can be maintained at a wide voltage range (Supplementary Fig. 23). Moreover, the intensity of current density at 30 mA cm^−2^ remained unchanged over the course of electrolysis (Fig. 4d, e), which is of great significance for highly efficient H_2_O_2_ generation in practical applications. Within 1 h at a current density of 30 mA cm^-2^, ~867 ppm H_2_O_2_ was generated with an average selectivity of ~92%. As a practical application, we applied the O–C(Al) catalyst to paper and pulp bleaching, and simulated the industrial experiment with blue litmus paper. The experiment showed that the produced H_2_O_2_ could almost completely destroy the existing colored material in the blue litmus paper, indicating that this scheme can potentially be applied to industrial applications (inset in Fig. 4e, Supplementary Fig. 24).

## Discussion

In summary, with the guidance of theoretical predictions, we have rationally constructed a series of oxygen-rich carbons with atomically dispersed Lewis acid sites, through the pyrolysis of MIL-53 with different metal clusters. Besides the inherited structural advantages of the MOF, the enriched atomically dispersed octahedral MO_6_ clusters in MOF-derived O–C(M) catalysts significantly improved the selectivity of O_2_ reduction to H_2_O_2_ by promoting the formation of *OOH and weakening the binding strength between the formed *OOH group and the carbon material. As a result, the catalytic performance of O–C(M) for reducing O_2_ to H_2_O_2_ is much higher than that of O–C in both alkaline and neutral media. More importantly, the increase in catalytic selectivity correlates well with Lewis acidity, following a nearly linear correlation. We envision that the current strategy, on forming atomically dispersed Lewis acid sites on the carbon matrix, can open an avenue to fabricate important high-performance future catalysts for 2-electron ORR and other similar processes.

## Methods

### Materials and characterization

All chemicals were obtained from commercial suppliers at analytical grade and used as received without further purification. Al(NO_3_)_3_ ∙ 9H_2_O, Ga(NO_3_)_3_ ∙ *x*H_2_O, Cr(NO_3_)_3_ ∙ 9H_2_O, aqueous HF, melamine, 1,10-phenanthroline, sulfur powder were obtained from Shanghai Aladdin Bio-Chem Technology Co., LTD. 1,4-benzene dicarboxylic acid was obtained from TCI Shanghai. Nafion solution was obtained from Sigma-Aldrich. Power X-ray diffractions (PXRD) patterns of the samples were collected on a D8-Advance Bruker AXS diffractometer with Cu kα (*λ* = 1.5418 Å) radiation at room temperature. Inductively coupled plasma mass spectroscopy (ICP-MS) measurements were carried on NexION 300 (PerkinElmer). The samples' morphologies were examined using a field emission scanning electron microscope (SEM, Hitachi, S-4800). Transmission electron microscopy (TEM) images were recorded on Tecnai F20 microscope. The aberration-corrected HAADF-STEM measurements were taken on a JEM-ARM200F instrument at 200 keV. X-ray photoelectron spectroscopy (XPS) measurements were performed by using a thermo ESCALAB 250 high-performance electron spectrometer using monochromatized Al Ka (*h*ν = 1486.6 eV) as the excitation source. Al K-edge X-ray absorption spectroscopy (XAS) was conducted at beamline 02B02 of the SiP·ME2 platform at the Shanghai Synchrotron Radiation Facility (SSRF).

### Preparation of MIL-53(Al)

MIL-53(Al) was synthesized and purified according to the reported procedures^33^. A typical synthesis involves a solution containing Al(NO_3_)_3_ ∙ 9H_2_O (1.3 g), 1,4-benzene dicarboxylic acid (0.288 g), de-ionized water (5 mL). The mixture was introduced into a Teflon-lined autoclave and heated for 3 d at 220 °C in an oven under static condition. The obtained MIL-53(Al) was purified twice by in ethanol at reflux temperature for 12 h and washed with hot ethanol. The sample was finally dried at 60 °C for 12 h under a dynamic vacuum prior to further use.

### Preparation of MIL-53(Ga)

MIL-53(Ga) was synthesized and purified according to the reported procedures^34^. A typical synthesis involves a solution containing Ga(NO_3_)_3_ ∙ *x*H_2_O (1.0 g), 1,4-benzene dicarboxylic acid (0.76 g), de-ionized water (20 mL). The mixture was introduced into a Teflon-lined autoclave and heated for 4 h at 210 °C in an oven under static condition. The obtained MIL-53(Ga) was purified twice by in ethanol at reflux temperature for 12 h and washed with hot ethanol. The sample was finally dried at 60 °C for 12 h under a dynamic vacuum prior to further use.

### Preparation of MIL-53(Cr)

MIL-53(Cr) was synthesized and purified according to the reported procedures^35^. A typical synthesis involves a solution containing Cr(NO_3_)_3_ ∙ 9H_2_O (1.384 g), 1,4-benzene dicarboxylic acid (0.575 g), de-ionized water (24 mL), aqueous HF (0.14 mL). The mixture was introduced into a Teflon-lined autoclave and heated for 3 d at 220 °C in an oven under static condition. The obtained MIL-53(Cr) was purified twice by in ethanol at reflux temperature for 12 h and washed with hot ethanol. The sample was finally dried at 60 °C for 12 h under a dynamic vacuum prior to further use.

### Preparation of O–C

Typically, 600 mg 1,4-benzene dicarboxylic acid was mixed with 30 mg carbon black and ground at room temperature for 1 h. Then the mixture was placed in a porcelain boat with a cap and charged into a flow-through tube furnace. The furnace was heated to 800 °C under a nitrogen atmosphere with a flow rate of 60 mL min^−1^, and then maintained at the target temperature for 2 h, followed by cooling down to room temperature in a nitrogen atmosphere.

### Preparation of O–C(M) (M = Al, Ga, Cr)

Typically, 600 mg MIL-53(Al, Ga, Cr) was placed in a porcelain boat and charged into a flow-through tube furnace. The furnace was heated to 800 °C under a nitrogen atmosphere with a flow rate of 60 mL min^−1^, and then maintained at the target temperature for 2 h, followed by cooling down to room temperature in a nitrogen atmosphere. The obtained solid was immersed in 10 mL aqueous solution of NaOH (0.1 M) and 1 mL ethanol with continued shaking at room temperature for 12 h. The final product was collected by centrifugation and washed 3 times with ethanol.

### Preparation of N–C

Typically, 2 g melamine and 50 mg carbon black were mixed with 2 mL ethanol solution of 1,10-phenanthroline (0.6 mmol) and 2 mL H_2_O, then the mixture was ground at room temperature for 1 h and then dried at 60 °C for 2 h under vacuum. The obtained solid was pyrolyzed at 600 °C for 2 h under a nitrogen atmosphere with a flow rate of 60 mL min^−1^. Finally, the resultant composite was placed in a porcelain boat with a cap and charged into a flow-through tube furnace. The furnace was heated to 800 °C under a nitrogen atmosphere with a flow rate of 60 mL min^−1^, and then maintained at the target temperature for 2 h, followed by cooling down to room temperature in a nitrogen atmosphere.

### Preparation of N–C(M) (M = Al, Ga)

Typically, 2 g melamine was mixed with 2 mL ethanol solution of 1,10-phenanthroline (0.6 mmol) and 2 mL aqueous solution of Al(NO_3_)_3_ or Ga(NO_3_)_3_ (0.2 mmol), then the mixture was ground at room temperature for 1 h and then dried at 60 °C for 2 h under vacuum. The obtained solid was pyrolyzed at 600 °C for 2 h under a nitrogen atmosphere with a flow rate of 60 mL min^−1^. Finally, the resultant composite was placed in a porcelain boat with a cap and charged into a flow-through tube furnace. The furnace was heated to 800 °C under a nitrogen atmosphere with a flow rate of 60 mL min^−1^, and then maintained at the target temperature for 2 h, followed by cooling down to room temperature in a nitrogen atmosphere.

### Preparation of S–C

First, 1 g carbon black was immersed in 40 mL aqueous solution of HCl (6 M) with continued to stir at 100 °C for 24 h, and the solid was washed three times with de-ionized water to remove all the possible residual acid. Then the solid was mixed with 20 g sulfur powder and ground at room temperature for 1 h. Finally, the mixture was placed in a porcelain boat with a cap and charged into a flow-through tube furnace. The furnace was heated to 800 °C under a nitrogen atmosphere with a flow rate of 60 mL min^−1^, and then maintained at the target temperature for 2 h, followed by cooling down to room temperature in a nitrogen atmosphere.

### Preparation of S–C(M) (M = Al, Ga)

Typically, 200 mg S–C was mixed with 1 mL aqueous solution of Al(NO_3_)_3_ or Ga(NO_3_)_3_ (0.43 mmol), then the mixture was ground at room temperature for 1 h and then dried at 60 °C for 2 h under vacuum. The obtained solid was placed in a porcelain boat with a cap and charged into a flow-through tube furnace. The furnace was heated to 800 °C under a nitrogen atmosphere with a flow rate of 60 mL min^−1^, and then maintained at the target temperature for 2 h, followed by cooling down to room temperature in a nitrogen atmosphere.

### DFT calculation

Density functional theory was performed with the Vienna ab initio simulation package (VASP)^53^. The ion–electron interaction was described by employing the projector augmented wave (PAW) method^54^. The exchange-correlation energy functional was described by the generalized gradient approximation (GGA) in the Perdew–Burke–Ernzerhof (PBE) functional^55^. The kinetic cutoff energy for the plane wave was set as 450 eV for all computational calculations. The convergence criteria for each atom were set to be 1E−5 eV for residual energy and 0.03 eV Å^−1^ for force. A 5 × 5 × 1 supercell of the graphene was employed as the simulation model. The vacuum space was 20 Å to avoid artificial interactions between periodic images. The k-points in the Brillouin zone were sampled by a 3 × 3 × 1 grid. The DFT-D3 method was used to describe the van der Waals (vdW) interactions between the adsorbed species and the catalyst. All the studied models are shown in Fig. 1 and Supplementary Fig. 1a, and the structures of the two possible reaction pathways are depicted in Supplementary Fig. 1b.

The computational hydrogen electrode (CHE) model^56^ was used to calculate the Gibbs reaction-free energy change (Δ*G*) for each step in the two-electron oxygen reduction reaction (ORR). According to the CHE model, the Δ*G* value was gained by Eq. (3):

3 $${\Delta}{{G}} = {\Delta}{{E}}_{{\mathrm{DFT}}} + {\Delta}{{E}}_{{\mathrm{ZPE}}} - {{T}}{\Delta}{{S}} + {{eU}} + {\Delta}{{G}}_{{\mathrm{pH}}},$$

where Δ*E*_DFT_ is the reaction energy, Δ_ZPE_ and Δ*S* are the changes in zero-point energy and entropy at 298.15 k, respectively. *T*, *e*, and *U* are the temperature, the number of electrons transferred and the electrode potential, respectively. The Δ*G*_pH_ is the free energy correction of pH, which can be calculated by Eq. (4):

4 $${\Delta}{{G}}_{{\mathrm{pH}}} = {\mathrm{ln}}10 \times {{k}}_{\mathrm{B}}{{T}} \times {\mathrm{pH}}.$$

The *k*_B_ is the Boltzman constant. Since RHE is taken as the reference, the pH was set to be zero in this calculation. The limiting potential (*U*_L_) is defined as the lowest potential as at which all the reaction steps are downhill in free energy.

The total energy (*E*) and corresponding thermodynamic quantities, in eV, for free H_2_, H_2_O, H_2_O_2_ species, were listed in Supplementary Table 8^39^. Since O_2_ molecule is poorly described by standard DFT calculations, all the free energies were calculated by using the free energies of H_2_O (l) and H_2_ (g) as ref. ^30^. The free energies of O_2_ molecule and H_2_O_2_ molecules are determined by equilibrium potential of four-electron (1.23 V vs. RHE) and two-electron (0.70 V vs. RHE) ORR, which are estimated by 4.92 (4 × 1.23) and 3.52 (4 × 1.23–0.70 × 2) eV, respectively.

For the two-electron ORR, there are two reaction steps^25^:

5 $$\ast + {\mathrm{O}}_2 + {\mathrm{H}}^ + + {{e}}^ - \to \ast {\mathrm{OOH}},$$

6 $$\ast {\mathrm{OOH}} + {\mathrm{H}} ^ ++ {{e}}^ - \to {\mathrm{H}_2}{\mathrm{O}_2} + \ast.$$

For the hydrogenation of oxygen (Eq. (5)), the Δ*G* was calculated by Eqs. (7)–(9):

7 $${\Delta}{{G}}_1 = {{G}}\left( { \ast {\mathrm{OOH}}} \right) - {{G}}\left( \ast \right) - {{G}}\left( {{\mathrm{O}}_2} \right) - 0.5 \times {{G}}\left( {{\mathrm{H}}_2} \right),$$

8 $${\Delta}{{G}}_1 = {{G}}\left( \ast {\mathrm{OOH}} \right) - {{G}}\left( \ast \right) - \left(4.92 - 2 \times {{G}}\left( {{\mathrm{H}}_2} \right) + 2 \times{{G}}\left( {{\mathrm{H}}_2{\mathrm{O}}} \right) \right)- 0.5 \times {{G}}\left( {{\mathrm{H}}_2} \right),$$

9 $${\Delta}{{G}}_1 = {{G}}\left( { \ast {\mathrm{OOH}}} \right) - {{G}}\left( \ast \right) - 4.92 + 1.5 \times {{G}}\left( {{\mathrm{H}}_2} \right) - 2 \times {{G}}\left( {{\mathrm{H}}_2{\mathrm{O}}} \right),$$

while for the reduction of OOH* to from H_2_O_2_ (Eq. (6)), the Δ*G* can be obtained directly by Eq. (10):

10 $${\Delta}{{G}}_2 = - 1.40\,{\mathrm{eV}} - {\Delta}{{G}}_1,$$

herein, the *G*(*OOH) is defined by Eq. (11):

11 $${{G}}\left( { \ast {\mathrm{OOH}}} \right) = 4.92\,{\mathrm{eV}} + {\Delta}{{G}}_1.$$

### The ORR activity and selectivity evaluated over a rotating ring-disk electrode

In general, the electrodes were prepared by dispersing the 3.3 mg catalyst and 30 µL Nafion in 1 mL ethanol. After sonication for 2 h, 6 µL of the catalyst ink was drop-dried onto a glassy carbon disk (area: 0.2475 cm^2^). The electrochemical tests were performed in a computer-controlled potentiostat (CHI-Instrument) with a four-electrode cell at room temperature. The rotating ring-disk electrode loaded with catalyst was used as the working electrode. A graphite rod and a saturated Ag/AgCl electrode were used as the counter and a reference electrode, respectively. Two electrolytes with pH ~ 13 (0.1 M NaOH) and ~7 (0.1 M phosphate-buffered saline and 0.5 M Na_2_SO_4_) were chosen. The ORR activity and selectivity were investigated by polarization curves and rotating ring-disk electrode measurements in oxygen saturated electrolyte at a scan rate of 10–20 mV s^–1^. A potential of 1.2 V (vs. RHE) was applied to the ring of the working electrode during the entire testing process.

H_2_O_2_ selectivity of catalysts on the rotating ring-disk electrode was calculated based on the current of the disk electrode and ring electrode. The expression is as Eq. (12):

12 $${\mathrm{Sel}}{\mathrm{.(\% ) = 2}} \times \frac{{{{I}}_{\mathrm{R}}{{/N}}}}{{{{I}}_{\mathrm{D}}+{{I}}_{\mathrm{R}}{{/N}}}},$$

*I*_R_: the ring current, *I*_D_: the disk current, *N*: the collection efficiency (0.35 after calibration).

### The ORR activity and selectivity evaluated over a H-cell system

In general, electrocatalytic H_2_O_2_ production on Teflon-treated carbon fiber paper loaded with catalysts (1 mg cm^–2^) was performed in a two-compartment cell with Nafion 117 membrane as a separator. Both the cathode compartment (20 mL) and anode compartment were filled with the same electrolyte. The H_2_O_2_ concentration was measured by a traditional titration method based on the mechanism that a colorless solution of Ti(SO_4_)_2_ would be oxidized by H_2_O_2_ to H_2_TiO_4_ with yellow color (Eq. (13)).

13 $${\mathrm{Ti}}^{4 + } + {\mathrm{H}}_2{\mathrm{O}}_2 + 2\,{\mathrm{H}}_2{\mathrm{O}} \to {\mathrm{H}}_2{\mathrm{TiO}}_4 + 4\,{\mathrm{H}}^ +.$$

Thus, the concentration of H_2_O_2_ after the reaction can be measured by UV-Vis spectroscopy. The wavelength used for the measurement of H_2_TiO_4_ was 408 nm.

The theoretical amount of H_2_O_2_ in 20 mL solution can be calculated via the consumed quantity of electric charge within the electrocatalysis. The expression is as Eq. (14):

14 $${{C}} 	= \frac{{{{Q}} \times {{M}}}}{{{\mathbf{2}} \times {\mathrm{NA}} \times {{Q}}_{\mathrm{e}} \times {{V}}}}= {{8}}{{.82}}\,{\mathrm{ppm}}\\ 	=\frac{{{{1{C}}} \times {{34 \ {\mathrm{g}} \ {\mathrm{mol}}^{-1}}}}}{{{{2}} \times {{6.02}} \times {{10^{23}{\mathrm{e}} \ {\mathrm{mol}}^{-1}}} \times {1.60} \times{10^{-19}} \ {C} \ {\mathrm{e}}^{-1} \times{20} \ {\mathrm{mL}}}}$$

NA: Avogadro constant (6.02 × 10^23^ e mol^−1^); *Q*: quantity of electric charge (*C*); *M*: relative molecular mass of H_2_O_2_ (34 g mol^−1^); *V*: the volume of the solution (20 mL); *C*: the concentration of H_2_O_2_ (g·mL^−1^); *Q*_e_: the quantity of electric charge for each electron (1.60 **×** 10^−19^ C·e^−1^).

## Supplementary information

Supplementary information

## Data Availability

The data that support the findings of this study are available from the corresponding author upon reasonable request.
