# Supplementary material for: Atomically dispersed Lewis acid sites boost 2-electron oxygen reduction activity of carbon-based catalysts
Source: Nat Commun. 2020 Oct 30;11:5478. doi: 10.1038/s41467-020-19309-4 (PMC7603490; doi:10.1038/s41467-020-19309-4)
Supplement: Supplementary file 1 — Supplementary information [file 41467_2020_19309_MOESM1_ESM.pdf]

Supplementary Information

Atomically Dispersed Lewis Acid Sites Boost 2-electron Oxygen Reduction Activity of Carbon-Based Catalysts

Qihao Yang et al.

This file includes:

Supplementary Figures

Supplementary Tables

Supplementary References

Supplementary Figures

The simulation models

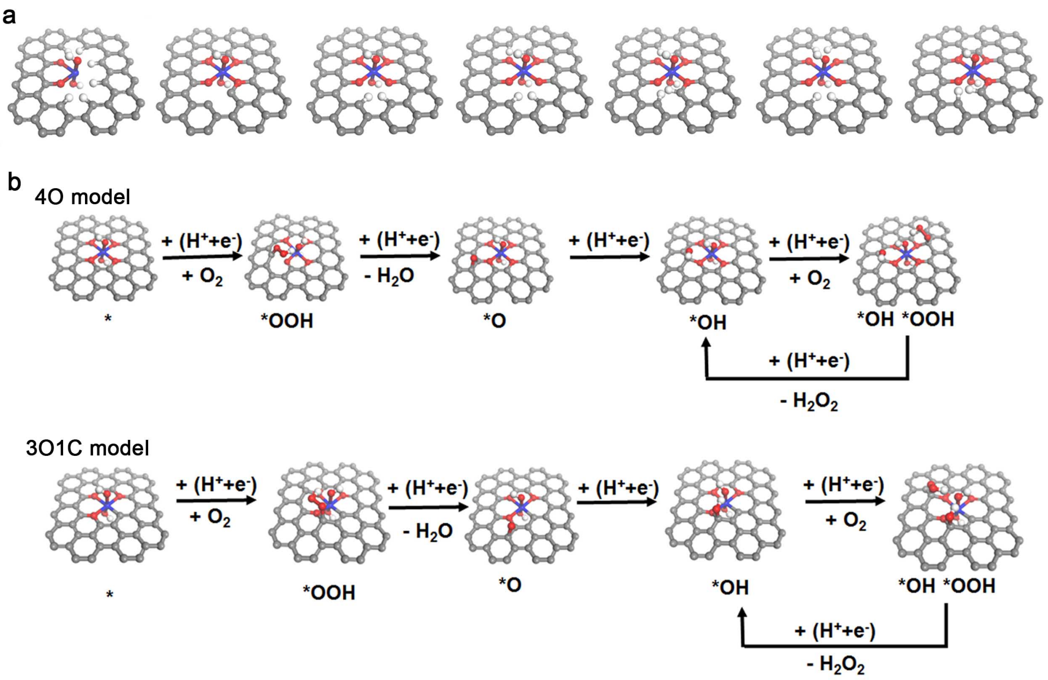

Supplementary Fig. 1. (a) The studied models containing terminal oxygen in simulation for electrochemical production of H_2O_2. Blue, red, white and gray balls denote metal, O, H and C atoms, respectively. (b) The structures of the key species of the two possible reaction pathways.

The energetic relationship

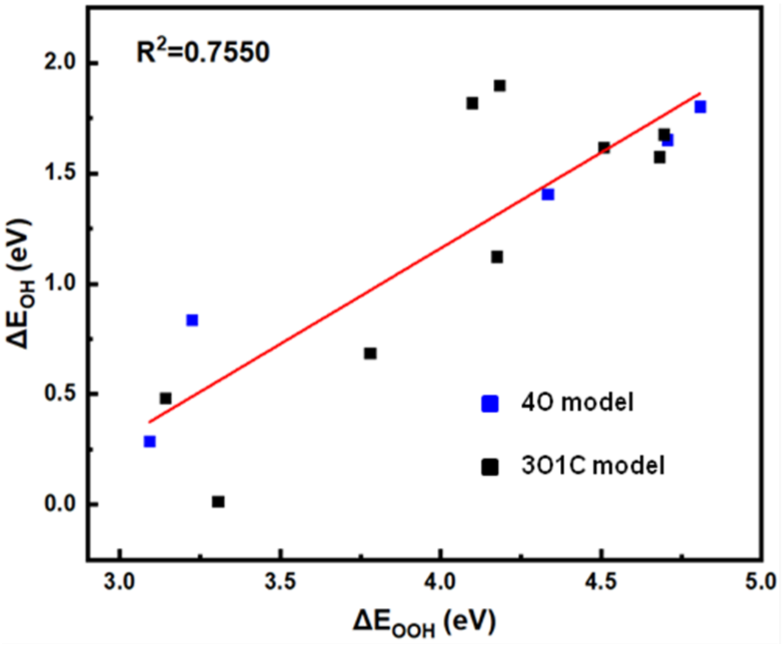

Supplementary Fig. 2. The relationship between computed energies of the first adsorbed *OOH (ΔE_{*OOH}) and *OH (ΔE_{*OH}) on various sites in 4O and 3O1C models.

The calculated deformation charge density

(a) (b)

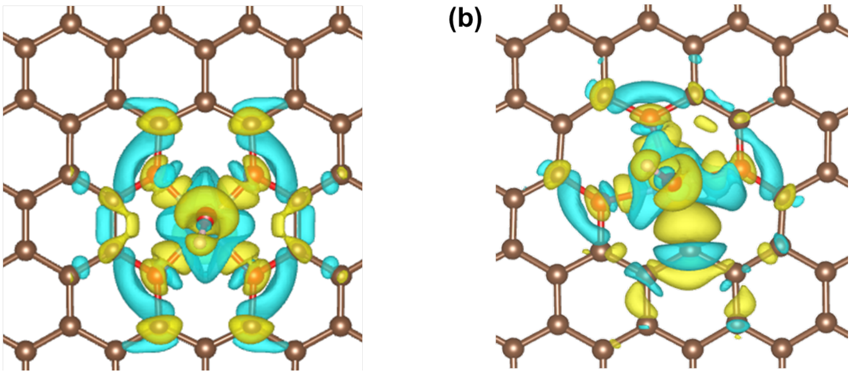

Supplementary Fig. 3. The deformation charge density between the oxygen-doped carbon layer and the metal motif with an isovalue of 0.0025 e/bohr^{-3}. (a) 4O model. (b) 3O1C model. Yellow and blue shadows represent electron accumulation and electron depletion, respectively.

The analysis results of the deformation charge density clearly show that the electron tends to accumulate on the α carbon after coordinating to Al or Ga.

The XRD Patterns

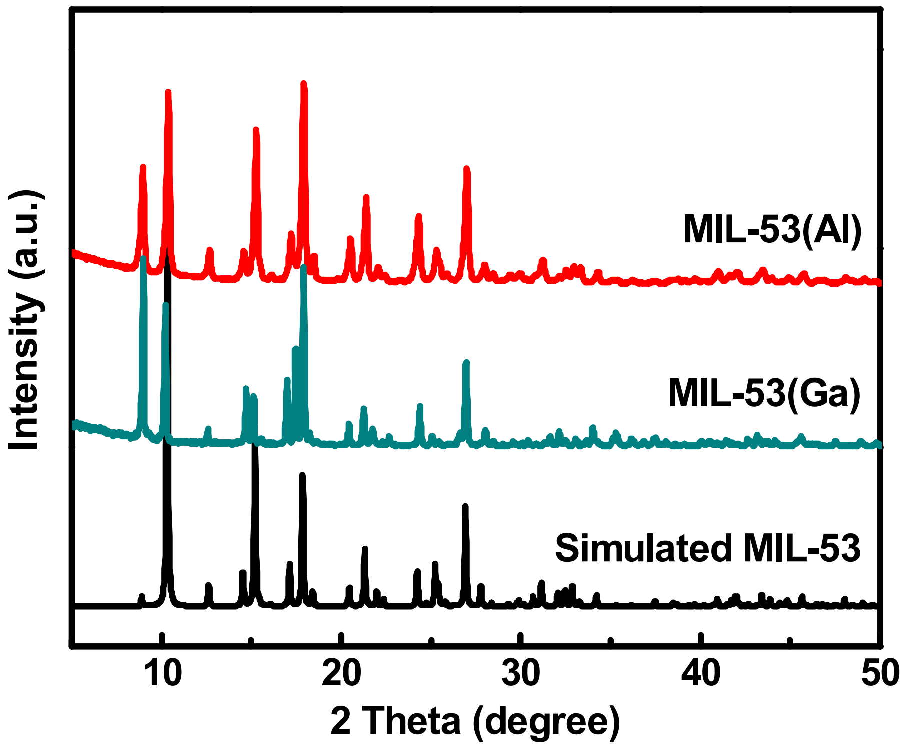

Supplementary Fig. 4. Powder XRD patterns for simulated MIL-53, as-synthesized MIL-53(Al), and MIL-53(Ga).

The SEM images

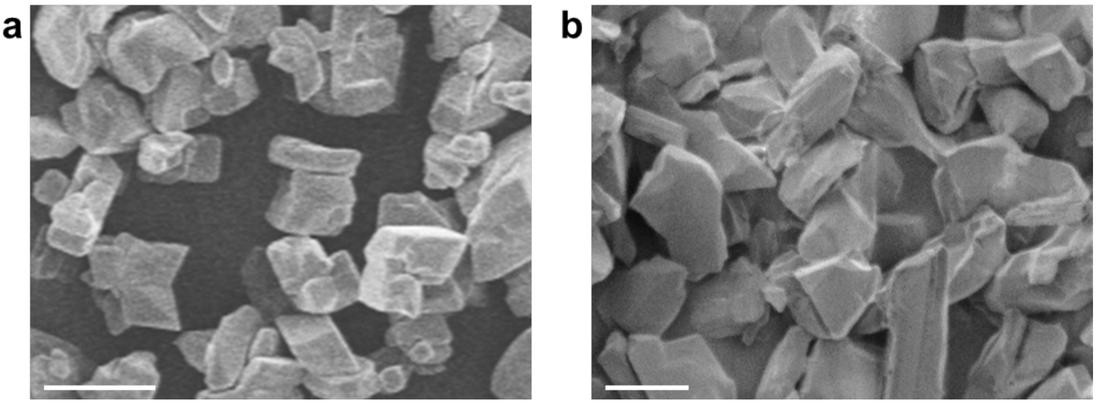

Supplementary Fig. 5. (a) SEM image for MIL-53(Al), scale bars are 5 μm. (b) SEM image for MIL-53(Ga), scale bars are 2 μm.

The SEM images

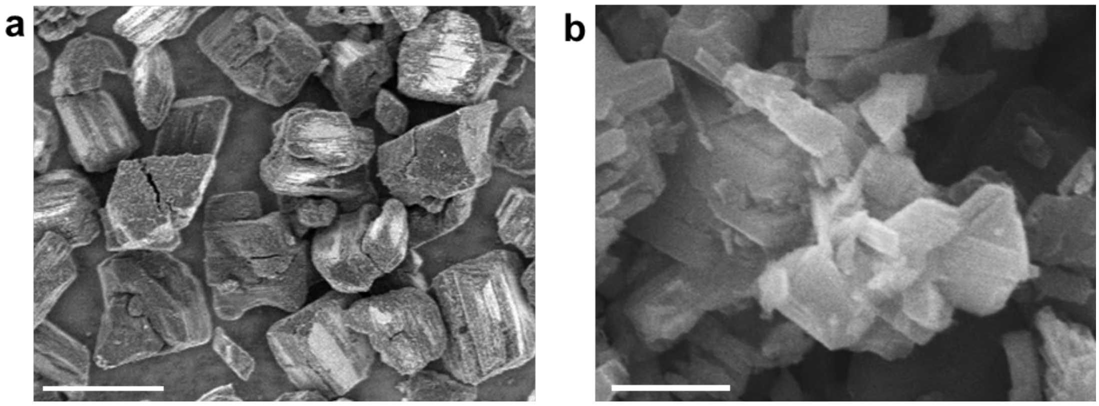

Supplementary Fig. 6. (a) SEM image for O-C(Al), scale bars are 5 μm. (b) SEM image for O-C(Ga), scale bars are 2 μm.

The SEM images

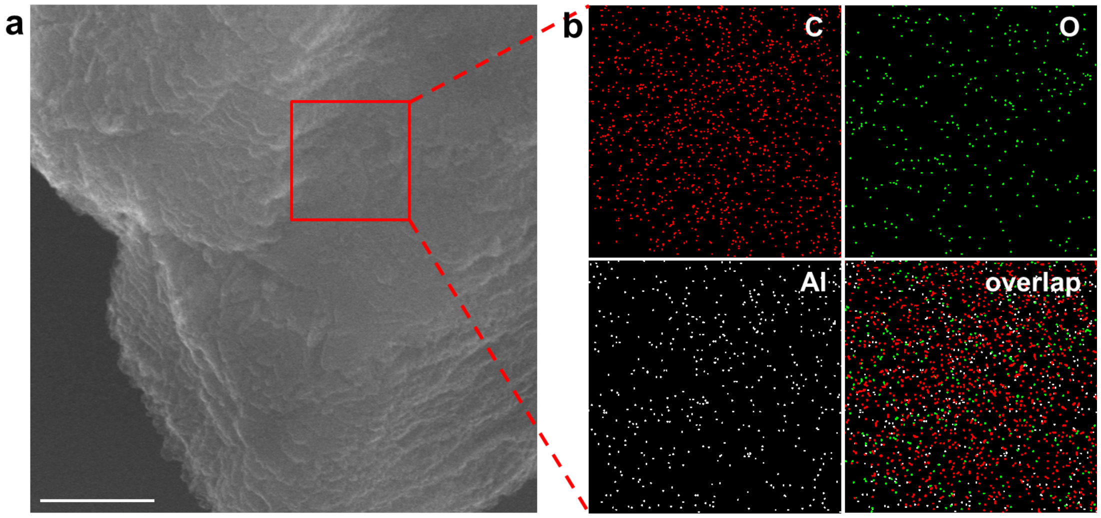

Supplementary Fig. 7. SEM images for (a) O-C(Al), scale bar is 500 nm. (b) The corresponding elemental mapping of C (red), O (green) and Al (white) for the selected area in (a).

The HAADF-STEM images

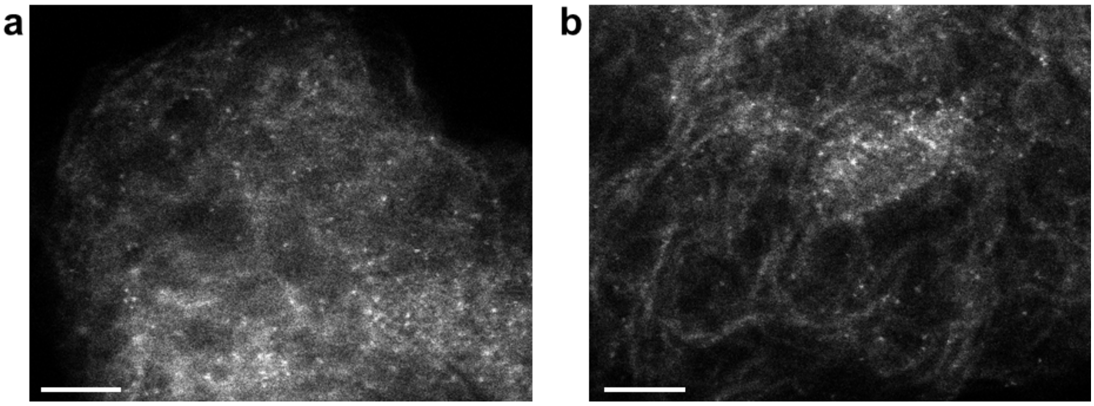

Supplementary Fig. 8. Aberration-corrected HAADF-STEM images of O-C(Ga) with different regions, scale bars are 2 nm.

The powder XRD patterns

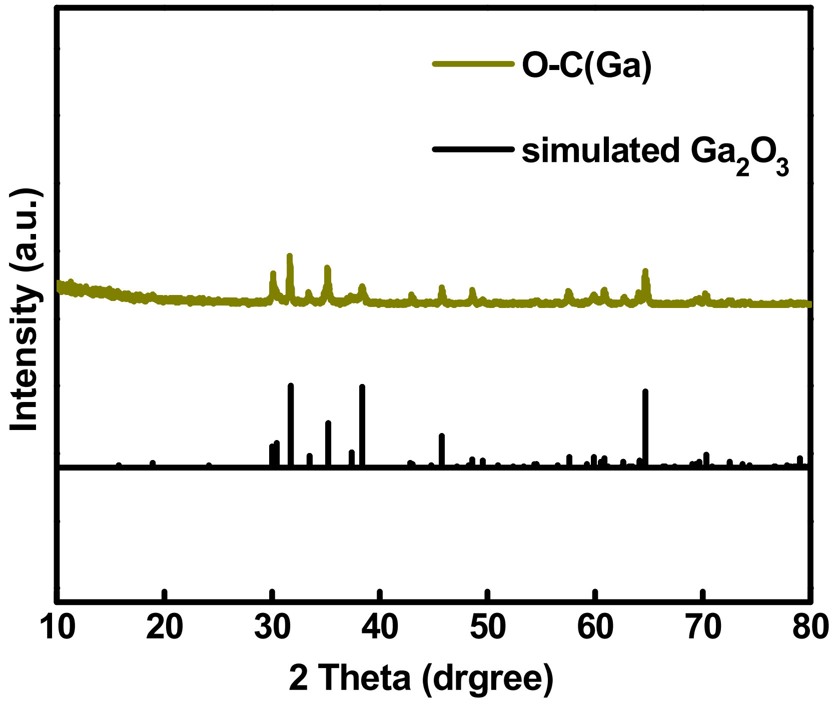

Supplementary Fig. 9. Powder XRD patterns for simulated Ga_2O_3 and synthesized O-C(Ga) obtained at 800 °C under N_2 atmosphere.

The XPS spectrum

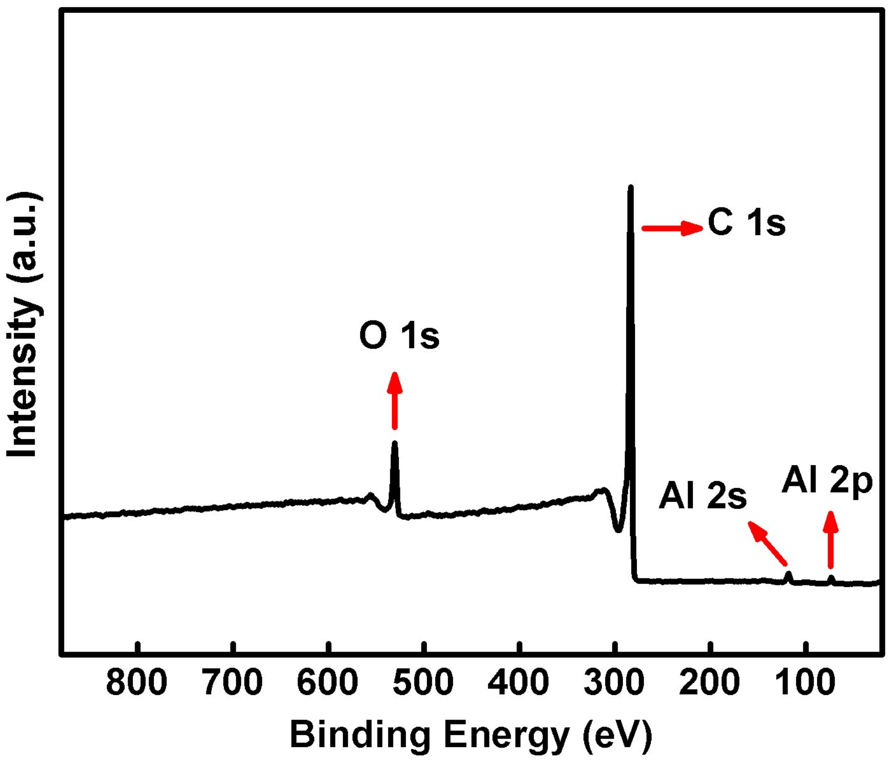

Supplementary Fig. 10. XPS survey spectrum for O-C(Al).

The XPS spectra

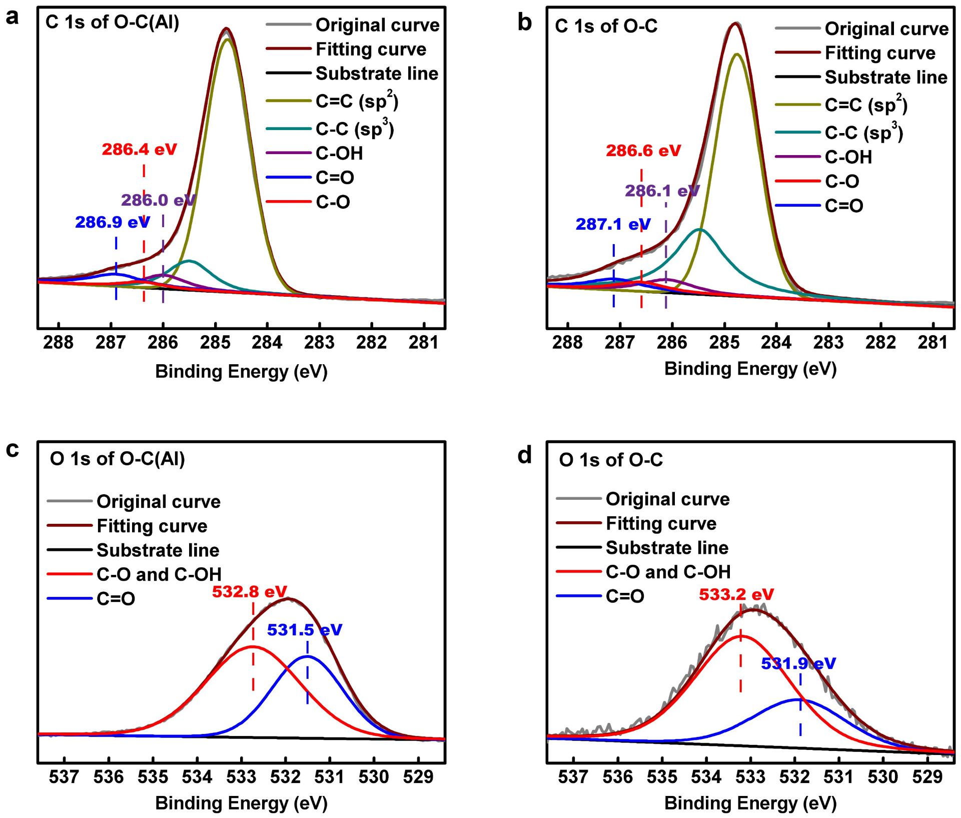

Supplementary Fig. 11. (a) High-resolution XPS spectrum of C 1s in O-C(Al). (b) High-resolution XPS spectrum of C 1s in O-C. (c) High-resolution XPS spectrum of O 1s in O-C(Al). (d) High-resolution XPS spectrum of O 1s in O-C.

The C 1s spectrum of O-C(Al) and O-C can be deconvoluted into the following bands: C=C (sp^2 carbon, 284.7 eV), C-C (sp^3 carbon, 285.5 eV), C-OH (hydroxyls, 286.0-286.1 eV), C-O (epoxides and ethers, 286.4-286.6 eV) and C=O (carbonyls and ketone, 286.9-287.1 eV). The deconvolution of the O 1s spectrum results in two peaks: oxygen doubly bound to carbon (i.e., C=O, 531.5-531.9 eV) and oxygen singly bound to carbon (i.e., O-C, 532.8-533.2 eV).[1-2]

The XANES results

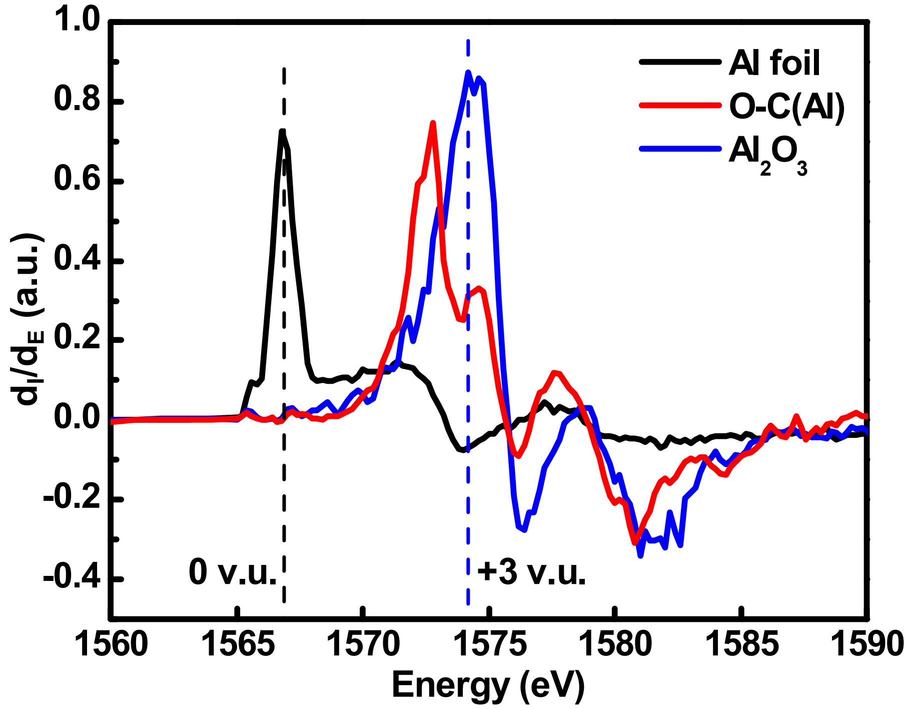

Supplementary Fig. 12. The differential curves of O-C(Al), Al_2O_3 and Al foils obtained from the corresponding XANES curves.

The differential curves show that the first peak position (inflection point, represent the valence of Al species) of O-C(Al) falls between those of Al foil and Al_2O_3, confirming that the valence of Al in O-C(Al) is indeed between 0 and +3.

The electrochemical measurements

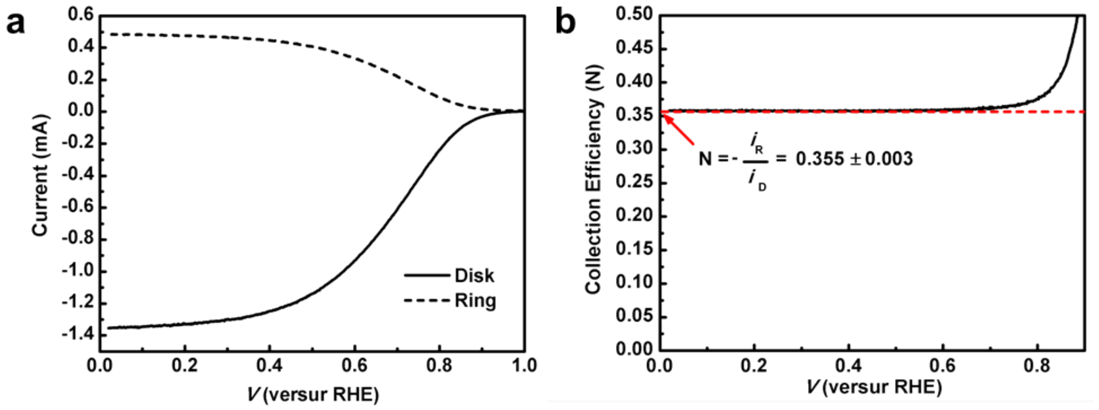

Supplementary Fig. 13. (a) Linear sweep voltammetry curves recorded on a bare glassy carbon rotation disk electrode with a Pt ring in the electrolyte containing 0.1 M KNO_3 and 10 mM $K_3Fe(CN)_6$ at 1600 rpm. Counter electrode: C rod, reference electrode: Ag/AgCl (0.2045 + 0.0592 × 7 = 0.6189), Ring potential: 1.2V vs RHE. (b) The experimental determined collection efficiency (N) via dividing the ring current by the disk ring in (a).

The powder XRD patterns

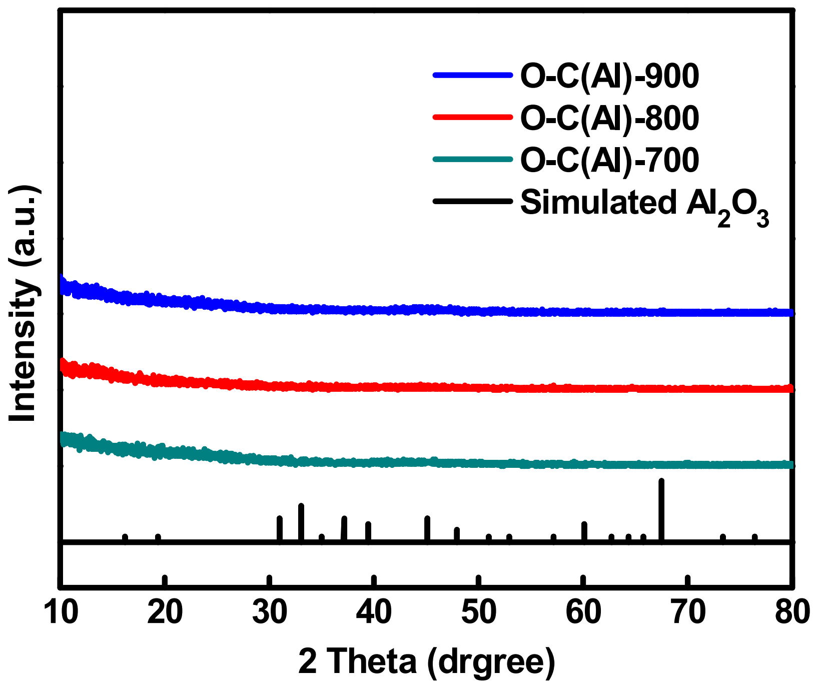

Supplementary Fig. 14. Powder XRD patterns for simulated Al_2O_3, as-synthesized O-C(Al) obtained at 700, 800 and 900 ºC (denoted as O-C(Al)-T, T = 700, 800 and 900 °C) under N_2 atmosphere.

The electrochemical measurements

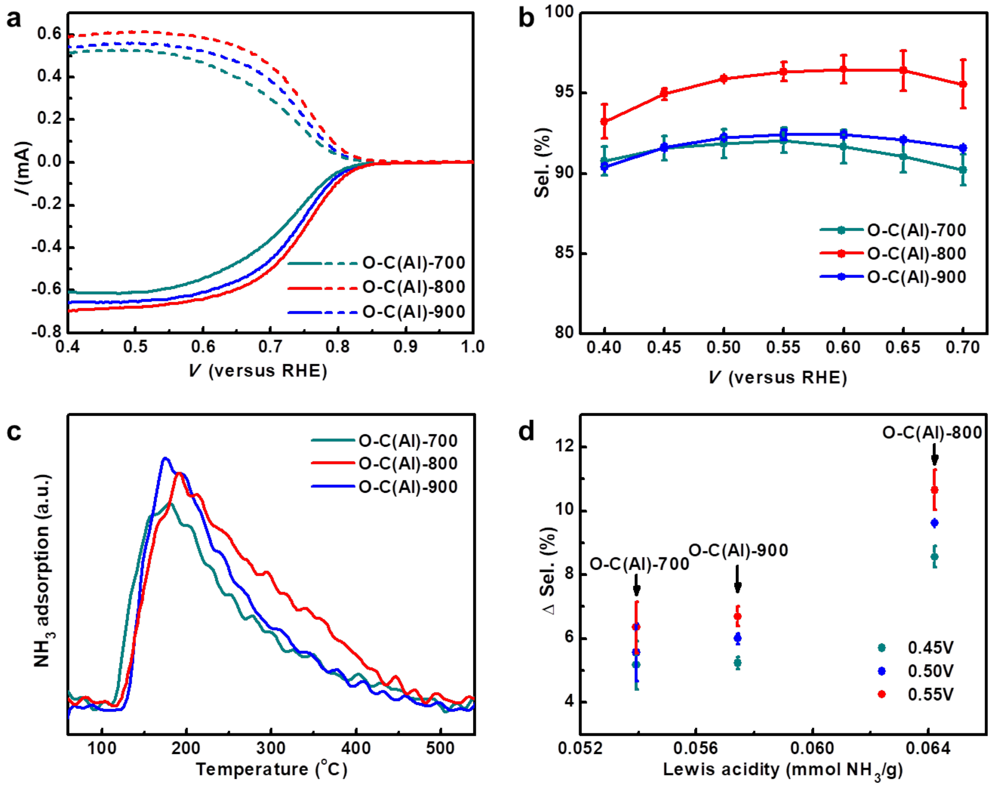

Supplementary Fig. 15. (a) LSV curves of O-C(Al) obtained at different temperatures (solid lines) together with the corresponding H_2O_2 currents on the ring electrode (dashed lines) recorded at 1600 rpm. (b) Calculated H_2O_2 selectivity during potential sweep. (c) NH_3-TPD profiles of O-C(Al) obtained at different temperatures. (d) The correlation between the increased selectivity (compared with O-C catalyst) and Lewis acidity of O-C(Al)-T (T = 700, 800 and 900 °C) catalysts at various potentials.

The powder XRD patterns and STEM image

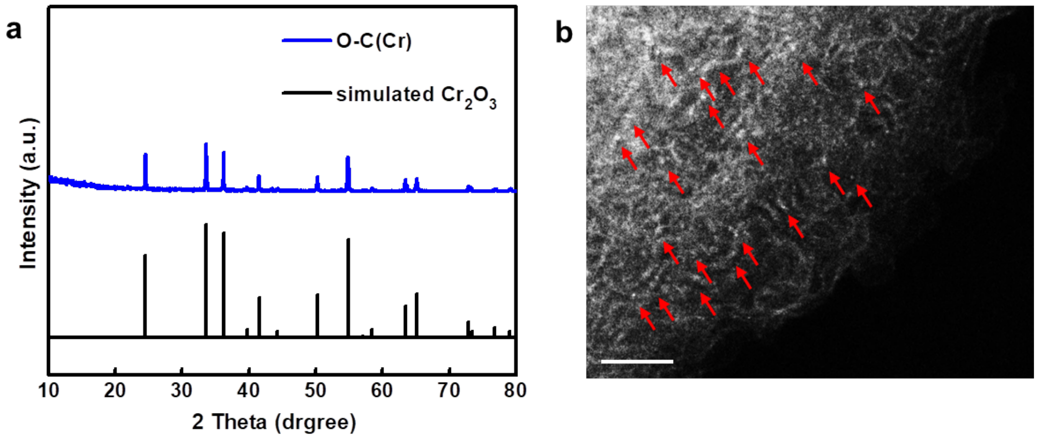

Supplementary Fig. 16. (a) Powder XRD patterns for simulated Cr_2O_3, as-synthesized O-C(Cr) obtained at 800 °C under N_2 atmosphere. (b) Aberration-corrected HAADF-STEM images of O-C(Cr), scale bar is 2 nm.

The electrochemical measurements

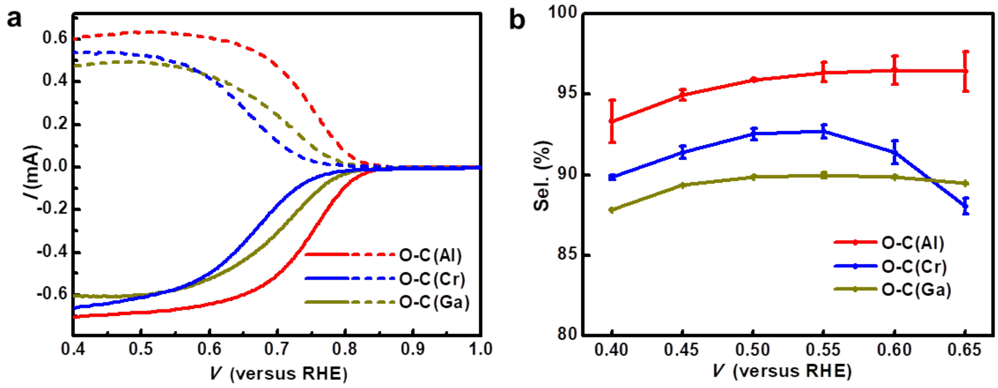

Supplementary Fig. 17. LSV curves of O-C(Al), O-C(Cr) and O-C(Ga) (solid lines) together with the corresponding H_2O_2 currents on the ring electrode (dashed lines) recorded at 1600 rpm. (b) Calculated H_2O_2 selectivity during potential sweep.

The electrochemical measurements

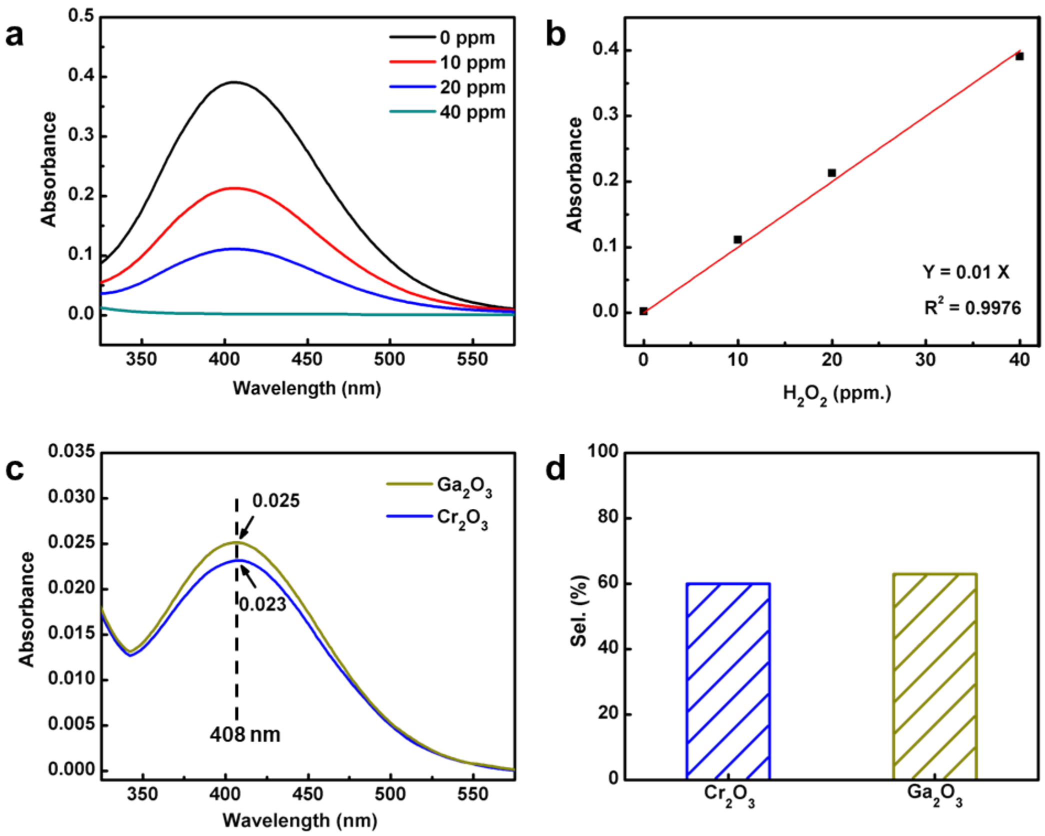

Supplementary Fig. 18. (a) UV-Vis absorption spectra for different concentration of H_2O_2 (1 mL) obtained in alkaline media. Titration solution (2 mL): 2 mM $Ti(SO_4)_2$ and 1 M H_2SO_4. (b) Relationship between the concentration of H_2O_2 and the absorbance of H_2TiO_4 at 408 nm in (a). (c) UV-Vis absorption spectra of the generated H_2O_2 solution with the catalysts of Ga_2O_3 and Cr_2O_3. The consumed quantity of electric charge within the electrocatalysis over Ga_2O_3 and Cr_2O_3 are 0.4472 C and 0.4374 C, respectively. Loading amount of catalyst: 1 mg·cm^{-2}, working potential: 0.45 V vs RHE, electrolyte: 0.1 M NaOH (20 mL). (d) Calculated H_2O_2 selectivity of Ga_2O_3 and Cr_2O_3.

The electrochemical measurements

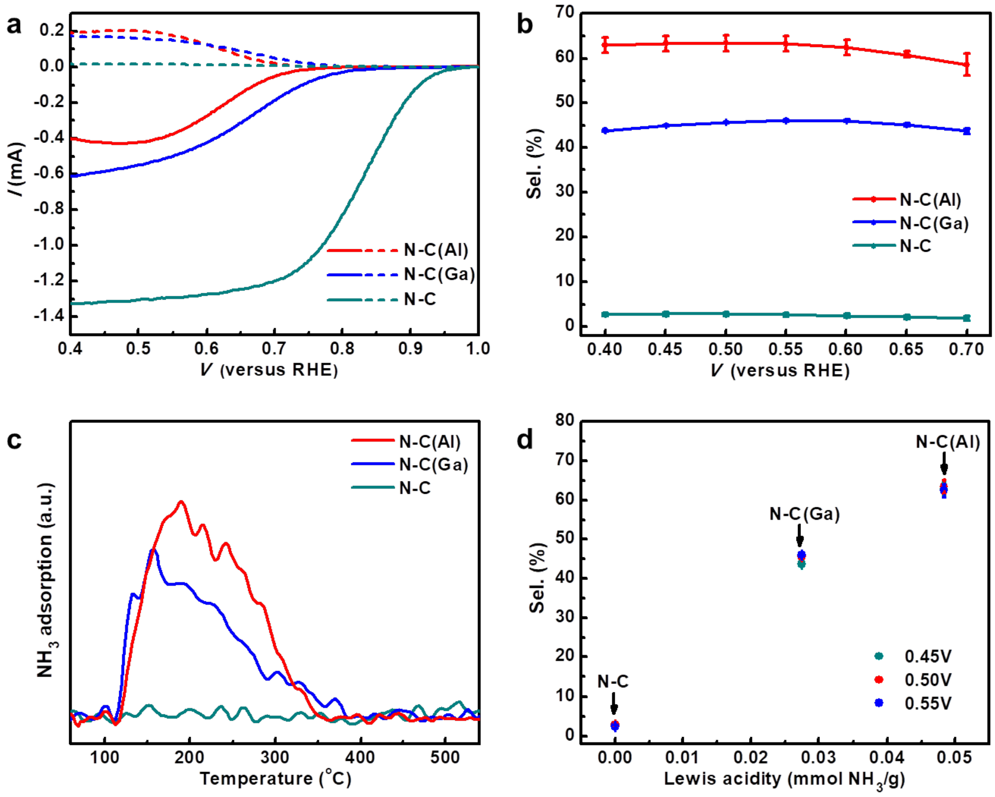

Supplementary Fig. 19. (a) LSV curves of N-C(M) (M = Al, Ga) and N-C (solid lines) together with the corresponding H_2O_2 currents on the ring electrode (dashed lines) recorded at 1600 rpm. (b) Calculated H_2O_2 selectivity during potential sweep. (c) NH_3-TPD profiles of N-C(M) and N-C obtained at different temperatures. (d) The correlation between the H_2O_2 selectivity and Lewis acidity of N-C(M) and N-C at various potentials.

The electrochemical measurements

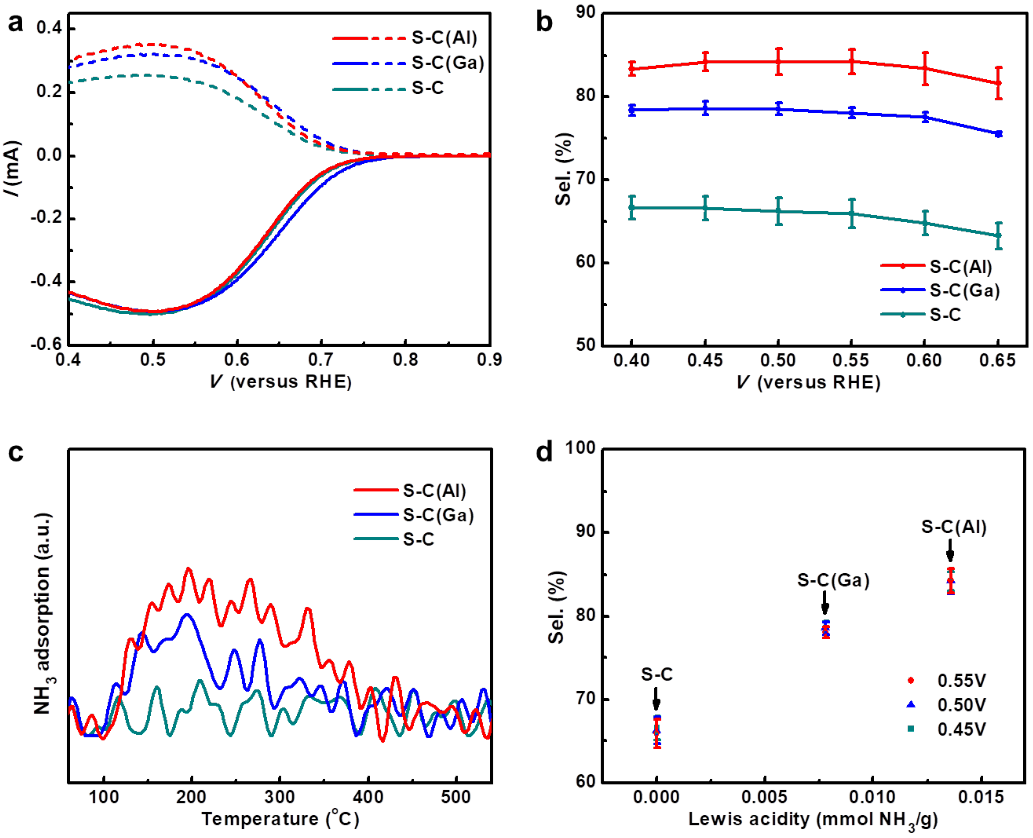

Supplementary Fig. 20. (a) LSV curves of S-C(M) (M = Al, Ga) and S-C (solid lines) together with the corresponding H_2O_2 currents on the ring electrode (dashed lines) recorded at 1600 rpm. (b) Calculated H_2O_2 selectivity during potential sweep. (c) NH_3-TPD profiles of S-C(M) and S-C obtained at different temperatures. (d) The correlation between the H_2O_2 selectivity and Lewis acidity of S-C(M) and S-C at various potentials.

The volcano plot

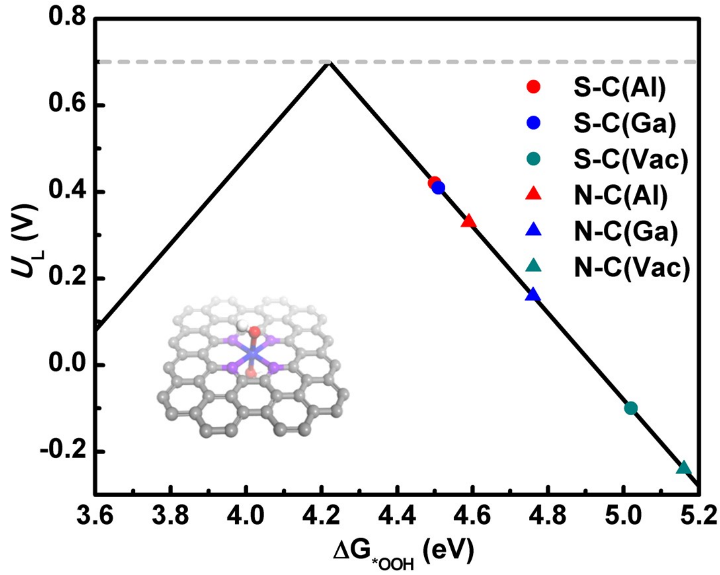

Supplementary Fig. 21. The volcano plot of S-C(M) and N-C(M) systems. Simulation model is shown in the inset in which the heteroatom is colored in purple.

The TPD results

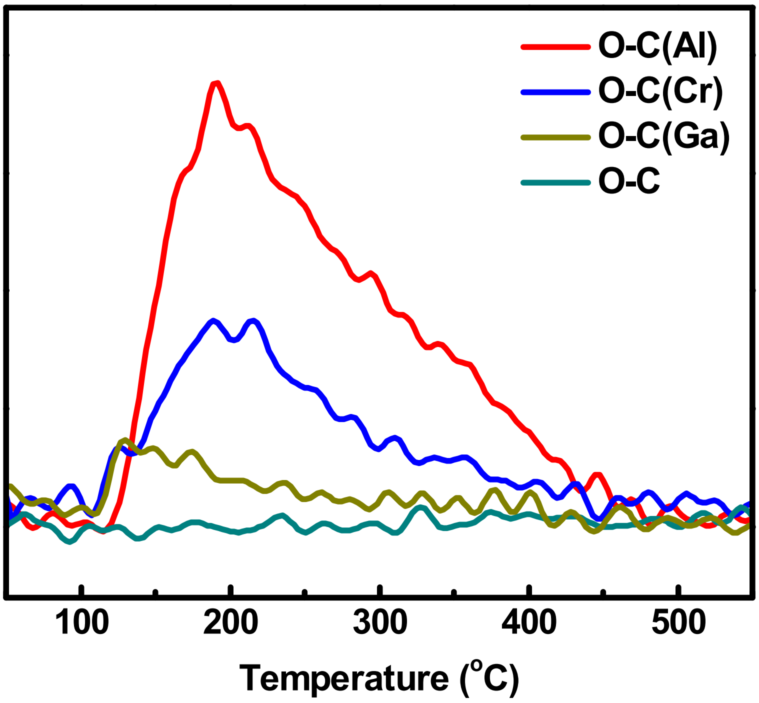

Supplementary Fig. 22. NH_3-TPD profiles of O-C(Al), O-C(Cr), O-C(Ga) and O-C.

The electrochemical measurements.

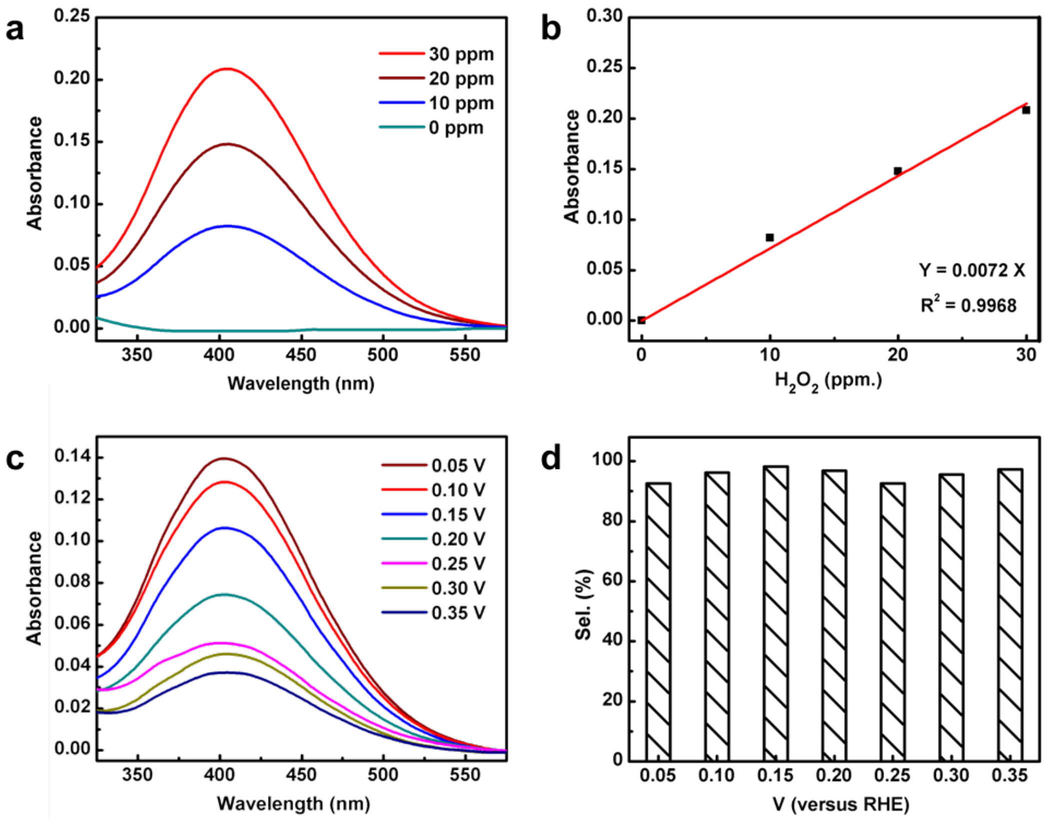

Supplementary Fig. 23. (a) UV-Vis absorption spectra for different concentration of H_2O_2 (1 mL) obtained in neutral media. Titration solution (1 mL): 1 mM $Ti(SO_4)_2$ and 1 M H_2SO_4. (b) Relationship between the concentration of H_2O_2 and the absorbance of H_2TiO_4 at 408 nm in (a). (c) UV-Vis absorption spectra of the generated H_2O_2 solution with the catalysts of O-C(Al) at different working potential. The consumed quantities of electric charge at 0.05-0.35 V vs RHE are 2.998 C, 2.361 C, 2.09 C, 1.697 C, 1.203 C, 0.865 C, 0.756 C and 0.6 C. Loading amount of catalyst: 1 mg·cm^{-2}, electrolyte: 0.5 M Na_2SO_4 (20 mL). (d) Calculated H_2O_2 selectivity of O-C(Al) in the neutral media.

The photographs

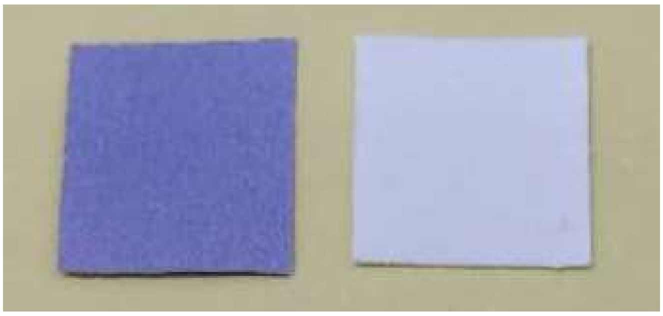

Supplementary Fig. 24. Photographs of blue litmus paper before (left) and after (right) bleaching with H_2O_2 prepared by electrocatalysis.

Supplementary Tables

Supplementary Table 1. The simulated |ΔG*OOH| data in the four discussed models.

Model	4O-Al	$4O_{OH}$-Al	3O1C-Al	$3O1C_{OH}$-Al	4O-Vac
ΔG_{*OOH} (eV)	3.48	4.00	3.52	4.19	4.99
Model	4O-Ga	$4O_{OH}$-Ga	3O1C-Ga	$3O1C_{OH}$-Ga	3O1C-Vac
ΔG_{*OOH} (eV)	3.10	3.76	3.51	3.98	4.63
Model	4O-Cr	$4O_{OH}$-Cr	3O1C-Cr	$3O1C_{OH}$-Cr	
ΔG_{*OOH} (eV)	3.15	3.78	3.66	4.04	

Supplementary Table 2. The calculated ΔG of the *OH desorption step of most favorable O_2 adsorption sites in metal-doped systems under external potential of 0.70 V.

model	4O		3O1C	
metal	Al	Ga	Al	Ga
ΔG	+0.06	+0.18	+0.33	+0.01

*OH desorption step: $OH^* + H^+ + e^- \rightarrow * + H_2O$

Supplementary Table 3. The calculated potential barrier of reductive H_2O_2 desorption step and the hydrogenation step of OOH* to O*.

model	$4O_{OH}$		$3O1C_{OH}$	
metal	Al	Ga	Al	Ga
ΔG_1	-0.43	-0.19	-0.62	-0.41
ΔG_2	0.01	0.28	-0.64	-0.27

ΔG_1 represents free energy change of the reductive H_2O_2 desorption step:

$$*OOH + H^+ + e^- \rightarrow H_2O_2$$

ΔG_2 represents free energy change of the step of hydrogenation OOH* to O*:

$$*OOH + H^+ + e^- \rightarrow O* + H_2O$$

Supplementary Table 4. EXAFS data fitting result of O-C(Al).

Sample	Bond type	Coordination Number	R (Å)	$\sigma^2 \times 10^{-3}$ ($Å^2$)	R-factor (%)
C-Al-O	Al-O/C	6.2 ± 0.8	1.73 ± 0.02	6 ± 2	0.02

R, distance between absorber and backscatter atoms; σ^2, Debye-Waller factor to account for both thermal and structural disorders; R-factor (%) indicates the goodness of the fit.

Supplementary Table 5. The performance comparison of the reported catalysts for electrochemical H_2O_2 production in alkaline medium.

Cat.	Electrolyte	Onset potential (V)	Areal loading ($mg \cdot cm^{-2}$)	Mass activity at 0.7V vs RHE ($A_{H2O2} \cdot g_{cat}^{-1}$)	Selectivity at 0.7V vs RHE (%)	Reference
O-C(Al)	0.1 M NaOH	0.824	0.08	23.9	97	This work
O-C(Ga)	0.1 M NaOH	0.795	0.08	12.3	89	This work
O-C(Cr)	0.1 M NaOH	0.759	0.08	6.1	81	This work
Co_1-NG(O)	0.1 M KOH	~ 0.83	0.01	~ 102	~ 76	3
Fe-CNT	0.1 M KOH	0.822	0.1	~ 4.2	~ 95	4
Co-CNT	0.1 M KOH	~ 0.80	0.1	~ 2.5	~ 75	4
Co-POC-O	0.1 M KOH	0.84	0.1	~ 12.1	~ 84	5
oxidized CNTs	0.1 M KOH	0.75	0.1	~ 12.6	~ 89	6
F-mrGO	0.1 M KOH	0.78	0.01	~ 60	> 95	7

Supplementary Table 6. The selectivity of Fe/Co/Ni/Mn doped electrocatalysts for O_2 reduction to H_2O_2 at high over potential (0.4V vs RHE) in alkaline media.

Cat.	Electrolyte	Selectivity (%)	Reference
O-C(Al)	0.1 M NaOH	~ 93	This work
O-C(Ga)	0.1 M NaOH	~ 88	This work
O-C(Cr)	0.1 M NaOH	~ 90	This work
Fe-CNT	0.1 M KOH	~ 80	4
Fe-N-CNT	0.1 M KOH	~ 25	4
Co-CNT	0.1 M KOH	~ 65	4
Co_1-NG(O)	0.1 M KOH	~ 80	3
Co-N-C	0.1 M KOH	~ 60	8
Co-POC-O	0.1 M KOH	~ 80	5
Mn-CNT	0.1 M KOH	~ 40	4

Supplementary Table 7. The comparison of simulated S-C(M) and N-C(M) performance. The U_L value close to 0.70 V is related to the outstanding activity to $2e^-$ ORR.

model	S-C(Al)	S-C(Ga)	S-C(Vac)	N-C(Al)	N-C(Ga)	N-C(Vac)
U_L (V)	0.42	0.41	-0.10	0.33	0.16	-0.24

Supplementary Table 8. The total energy (E) and corresponding thermodynamic quantities.

Species	E	TS	ZPE	PBE-Correction	G
H_2 (g)	-6.76	0.40	0.27	-0.08	-6.97
H_2O (l)	-14.22	0.67	0.57	-0.06	-14.39
H_2O_2 (l)	-18.16	0.72	0.57	-0.09	-18.28

Supplementary References

1. Kundu S. Wang Y. Xia W. & Muhler M. Thermal stability and reducibility of oxygen-containing functional groups on multiwalled carbon nanotube surfaces: a quantitative high-resolution XPS and TPD/TPR study. *J. Phys. Chem. C* **112**, 16869-16878 (2008).
2. Kim H. W. *et al.* Efficient hydrogen peroxide generation using reduced graphene oxide-based oxygen reduction electrocatalysts. *Nat. Catal.* **1**, 282-290 (2018)
3. Jung E. et al. Atomic-level tuning of Co-N-C catalyst for high-performance electrochemical H_2O_2 production. *Nat. Mater.* **19**, 436-442 (2020).
4. Jiang K. et al. Highly selective oxygen reduction to hydrogen peroxide on transition metal single atom coordination. *Nat. Commun.* **10**, 3997 (2019).
5. Li B. Q., Zhao C. X., Liu J. N. & Zhang Q. Electrosynthesis of hydrogen peroxide synergistically catalyzed by atomic Co-N_x-C sites and oxygen functional groups in noble-metal-free electrocatalysts. *Adv. Mater.* **31**, 1808173 (2019).
6. Lu Z. Y. et al. High-efficiency oxygen reduction to hydrogen peroxide catalysed by oxidized carbon materials. *Nat. Catal.* **1**, 156-162 (2018).
7. Kim H. W. et al. Efficient hydrogen peroxide generation using reduced graphene oxide-based oxygen reduction electrocatalysts. *Nat. Catal.* **1**, 282-290 (2018).
8. Sun Y. Y. et al. Activity-selectivity trends in the electrochemical production of hydrogen peroxide over single-site metal-nitrogen-carbon catalysts. *J. Am. Chem. Soc.* **141**, 12372-12381 (2019).
